# Combining photodynamic therapy and cascade chemotherapy for enhanced tumor cytotoxicity: the role of CTT_2_P@B nanoparticles

**DOI:** 10.3389/fbioe.2024.1361966

**Published:** 2024-02-12

**Authors:** Rongyi Wang, Hongsen Li, Lu Han, Boao Han, Yiting Bao, Hongwei Fan, Chaoyue Sun, Ruijie Qian, Liying Ma, Jiajing Zhang

**Affiliations:** School of Pharmacy, Binzhou Medical University, Yantai, China

**Keywords:** mitochondria-targeting, photodynamic therapy, bovine serum albumin, camptothecin, ROS-responsive

## Abstract

The mitochondria act as the main producers of reactive oxygen species (ROS) within cells. Elevated levels of ROS can activate the mitochondrial apoptotic pathway, leading to cell apoptosis. In this study, we devised a molecular prodrug named CTT_2_P, demonstrating notable efficacy in facilitating mitochondrial apoptosis. To develop nanomedicine, we enveloped CTT_2_P within bovine serum albumin (BSA), resulting in the formulation known as CTT_2_P@B. The molecular prodrug CTT_2_P is achieved by covalently conjugating mitochondrial targeting triphenylphosphine (PPh_3_), photosensitizer TPPOH_2_, ROS-sensitive thioketal (TK), and chemotherapeutic drug camptothecin (CPT). The prodrug, which is chemically bonded, prevents the escape of drugs while they circulate throughout the body, guaranteeing the coordinated dispersion of both medications inside the organism. Additionally, the concurrent integration of targeted photodynamic therapy and cascade chemotherapy synergistically enhances the therapeutic efficacy of pharmaceutical agents. Experimental results indicated that the covalently attached prodrug significantly mitigated CPT cytotoxicity under dark conditions. In contrast, TPPOH_2_, CTT_2_, CTT_2_P, and CTT_2_P@B nanoparticles exhibited increasing tumor cell-killing effects and suppressed tumor growth when exposed to light at 660 nm with an intensity of 280 mW cm^−2^. Consequently, this laser-triggered, mitochondria-targeted, combined photodynamic therapy and chemotherapy nano drug delivery system, adept at efficiently promoting mitochondrial apoptosis, presents a promising and innovative approach to cancer treatment.

## 1 Introduction

Based on the most recent Global Cancer Statistics 2020 report, cancer has emerged as a primary or secondary factor contributing to mortality among individuals below the age of 70 in 112 nations worldwide (Sung et al., 2021). Chemotherapy, a conventional cancer treatment modality, often induces significant side effects due to its limited therapeutic specificity, bioavailability, and vulnerability to multidrug resistance (Li et al., 2018; Pan et al., 2021; Zou et al., 2021). Prodrugs provide a technique to accomplish targeted drug activation at the site of the tumor, liberating bioactive medications as needed in order to minimize overall toxicity. Prodrug activation mainly relies on utilizing distinct characteristics of the tumor microenvironment, such as reduced pH, glutathione concentrations, hypoxia, and specific enzyme expression. Additionally, external stimuli like light, heat, and ultrasound can also be employed for this purpose (Yin et al., 2019). Red and near-infrared light are attractive stimuli for prodrug activation due to their deep tissue penetration depth and low phototoxicity.

Phototriggered prodrugs have shown great potential in tumor treatment, given their non-invasive and remote spatiotemporal control capabilities, which are increasingly utilized in precision tumor treatment. When exposed to laser radiation, photosensitizers have the ability to produce significant amounts of reactive oxygen species (ROS) (O'Connor et al., 2009; Yi et al., 2018; Agwa et al., 2023; Yang et al., 2023; Yu et al., 2023). These ROS can eliminate cancer cells and break down ROS-sensitive chemical bonds, such as thioketal (TK) (Shi et al., 2018), alkyl thioether or selenide (Luo et al., 2019), aryl boronic acid or ester (Daum et al., 2017). Utilizing ROS-sensitive bonds to covalently link chemotherapeutic drugs and photosensitizers in the design of photo-triggered molecular prodrugs improves the specificity of chemotherapy drugs, reduces side effects, and synchronizes drug distribution *in vivo* (Liu et al., 2016; Xu et al., 2016; Jiang et al., 2020; Lebepe et al., 2021). However, despite allowing temporal and spatial control of drug release, diverse release sites within the cell can significantly impact drug therapeutic efficacy. Therefore, exploring strategies to optimize therapeutic outcomes is imperative.

Mitochondria, which are versatile organelles that control cellular bioenergy, calcium balance, redox equilibrium, and programmed cell death, are responsible for 90% of intracellular ROS generation (Nunnari and Suomalainen, 2012; Yue et al., 2016a; Xie et al., 2020). Mitochondria possess antioxidant systems to maintain redox homeostasis, and ROS at specific concentrations benefit normal cellular physiological functions. However, excessive ROS disrupts redox equilibrium, inducing mitochondrial death (Porporato et al., 2018; Wang et al., 2023). The mitochondrial genome lacks an effective DNA damage repair mechanism, making it a sensitive target for drugs that act on DNA and reverse chemotherapy resistance (Mallick et al., 2018). The close proximity of the photosensitizer to the target area greatly enhances the effectiveness of photodynamic therapy, as a result of the brief lifespan and restricted spread of ROS in biological systems (<0.1 ms, <20 nm) (Yuan et al., 2014). Delivering chemotherapeutic medicines and photosensitizers to mitochondria maximizes treatment efficacy.

Here, we developed CTT_2_P, a molecular prodrug targeting mitochondria. CTT_2_P comprises a triphenylphosphine cation, a porphyrin-type photosensitizer, a ROS-sensitive bond thioketal (TK), and covalently coupled camptothecin (CPT). During cancer treatment, CPT, a typical medicine for the mitochondrial apoptosis pathway, acts as an inhibitor of cellular respiration and DNA topoisomerase I (Liu et al., 2019; Zhang et al., 2019; Chen et al., 2023). CPT was chosen as a chemotherapeutic drug to promote the generation of ROS and induce cell apoptosis in synergism with the photosensitizer laser-induced dual pathway. Positively charged compounds are accumulated in the mitochondrial matrix of cancer cells due to their more negative membrane potential in the mitochondrial inner membrane compared to normal cells. With its excellent lipophilicity and positive charge, triphenylphosphine cation is a commonly utilized mitochondrial targeting group, facilitating rapid substance transport across the membrane to mitochondria (Mehta et al., 2017; Zielonka et al., 2017). Tumor cells, more sensitive to mitochondrial perturbations due to widespread metabolic reprogramming, benefit from prodrugs acting at the mitochondrial site, improving drug efficacy and efficiently promoting mitochondrial apoptosis, subsequently triggering cell apoptosis (Fu et al., 2019).

However, molecular prodrugs exhibit low solubility and are quickly metabolized in blood circulation, resulting in low bioavailability. Therefore, improving drug accumulation in tumor tissue becomes a practical consideration. Utilizing the enhanced permeability and retention (EPR) effect at the tumor site, nanoparticles (NPs) composed of either natural or synthetic substances efficiently administer a precise amount of molecular therapeutics to tumor tissue, proving to be an effective approach for drug delivery (Mayr et al., 2017; Luo et al., 2023).

**SCHEME 1:**
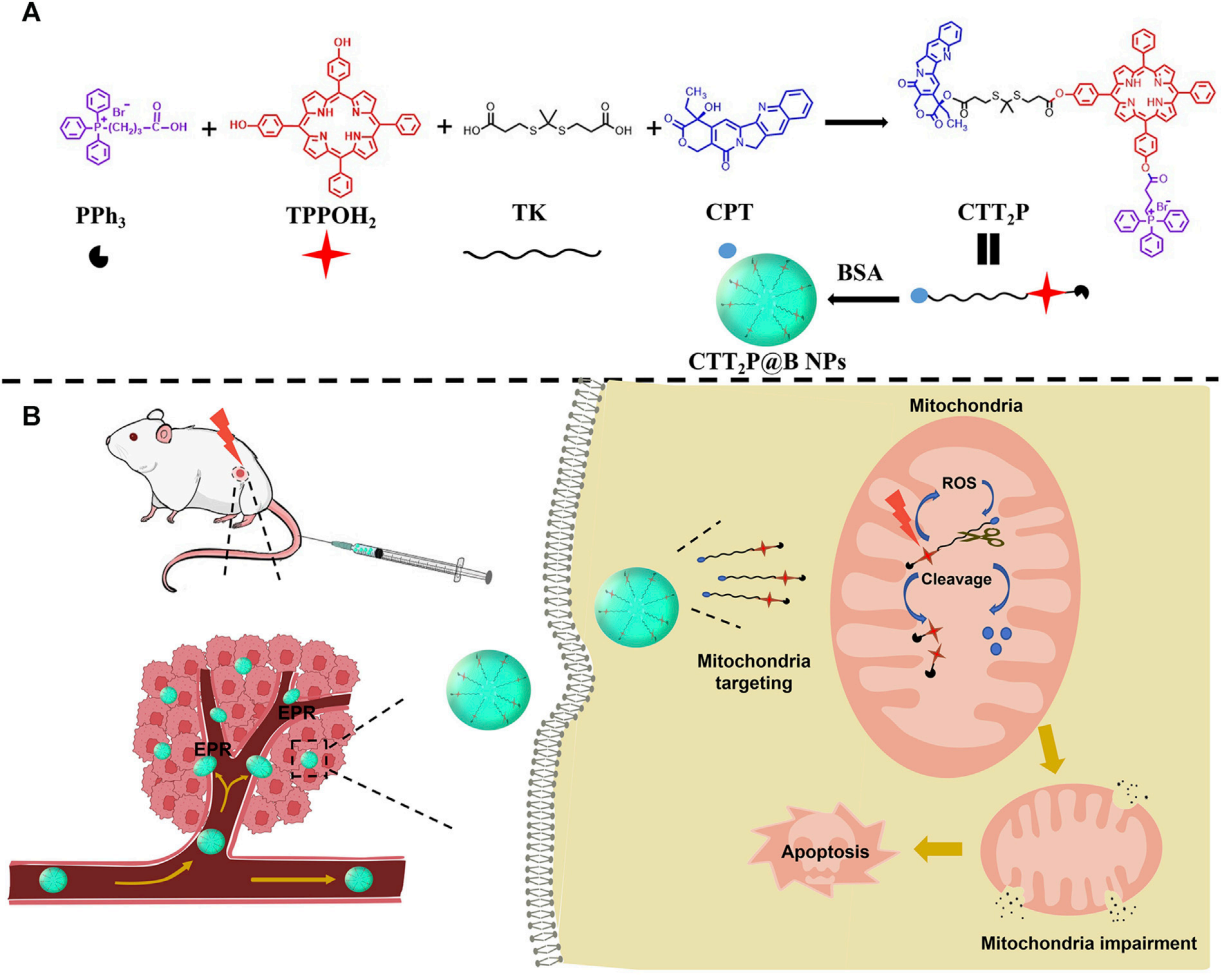
Design of mitochondria-targeted ROS-responsive nanomedicine. **(A)** Schematic of each part of the CTT_2_P prodrug-linked compound and its encapsulation into CTT_2_P@B NPs by BSA. **(B)** Schematic diagram of CTT_2_P NPs release and treatment at local tumor sites.

In this study, we created the molecular drug CTT_2_P targeting mitochondria for ROS responsiveness (Scheme 1A, B). We encapsulated it through bovine serum albumin (BSA) as CTT_2_P@BSA NPs to prolong drug circulation, delivering CTT_2_P to the tumor site for maximal therapeutic efficacy with minimal side effects. First, CTT_2_ was obtained by covalently linking the porphyrin derivative TPPOH_2_ to the chemotherapeutic drug CPT using the ROS-sensitive bond TK. Derivating the 20-hydroxy group of CPT can decrease activity, reducing the side effects of chemotherapy drugs. (Hong et al., 2000). Then, triphenylphosphine PPh_3_ was covalently linked to CTT_2_ to achieve mitochondrial targeting of CTT_2_P. Finally, a nano-drug delivery system (CTT_2_P@B) was constructed by coating CTT_2_P with bovine serum albumin (BSA). Triphenyl phosphate groups target CTT_2_P to mitochondria after CTT_2_P@B releases the prodrug CTT_2_P in tumor cells. Low levels of ROS in tumor cells are insufficient to cleave TK bonds (Yin et al., 2019; Chu et al., 2020). However, irradiation with a 660 nm laser causes the photosensitizer component to generate ROS, significantly increasing ROS levels and enhancing cell damage.

Additionally, it cleaves the TK bond, releasing the photosensitizer and effective CPT in the mitochondria. CPT accumulation in mitochondria leads to ROS production (Zhang et al., 2019), increasing the breakage of the TK link and the cumulative release of CPT. This repeated superposition self-cycling action boosts mitochondrial apoptosis and triggers cell apoptosis with high efficiency and low toxicity.

## 2 Material and methods

### 2.1 Materials

Camptothecin (CPT), 3-mercaptopropionic acid, p-hydroxybenzaldehyde, benzaldehyde, 4-dimethylaminopyridine (DMAP), and 1,3-diphenylisobenzofuran (DPBF) were purchased from Shanghai Macklin Biochemical Technology Co., Ltd. (Shanghai, China). Pyrrole, 1-(3-dimethylaminopropyl)-3-ethylcarbodiimide hydrochloride (EDCI), (3-carboxypropyl) triphenyl phosphonium bromide (PPh_3_), and Bovine Serum Albumin (BSA) were purchased from Shanghai Aladdin Biochemical Technology Co., Ltd. (Shanghai, China). Propionic acid was purchased from Anhui Zesheng Technology Co., Ltd. (Shanghai, China). Mito-Tracker Green, Reactive Oxygen Species Assay Kit (DCFH-DA) was purchased from Beyotime Biotechnology Co., Ltd. (Shanghai, China). Annexin-V FITC/PI, Mitochondrial Membrane Potential Assay Kit (with JC-1) was purchased from Elabscience Biotechnology Co., Ltd. (Hubei, China). All other reagents were of analytical grade and used as received.

### 2.2 Synthesis of TK

The synthesis of thioketal (TK) was carried out according to established procedures (Yue et al., 2016b). Anhydrous acetone (5.70 g, 98.20 mmol) and 3-mercaptopropionic acid (5.21 g, 49.10 mmol) were thoroughly mixed, and anhydrous hydrogen chloride gas was introduced. The reaction proceeded for 4 h at room temperature and was terminated. After crystallization in an ice water bath, the resulting product underwent filtration and was alternatively washed with hexane and cold water. Vacuum drying yielded the white powder product TK, with a 27. 3% yield. Confirmation of TK was achieved through ^1^H NMR spectroscopy (500 MHz, CDCl_3_): δ 2.90 (t, 4H), 2.68 (t, 4H), 1.59 (s, 6H).

### 2.3 Synthesis of 5, 10-bis (4-hydroxyphenyl)-15, 20-diphenyl porphyrin (TPPOH_2_)

For the synthesis of TPPOH_2_, p-hydroxybenzaldehyde (45.00 mmol, 5.50 g) and benzaldehyde (45.00 mmol, 4.57 mL) were added to 150 mL of propionic acid and heated to reflux. Within a time frame of 20 min, Pyrrole (90.00 mmol, 6.23 mL) was introduced into a constant pressure funnel. The mixture was then stirred at a temperature of 140°C for a duration of 90 min. Following the reaction, vacuum concentration removed the propionic acid, producing an oily liquid. The liquid was dissolved in a mixture of dichloromethane and methanol (in a ratio of 70:1), then filtered. The resulting solution was concentrated under vacuum to obtain the crude product. Separation and purification through silica gel column chromatography (100–200 mesh) with dichloromethane: methanol ratios of 50:1 and 100:1 yielded the purple product TPPOH_2_, with a 2.4% yield. The confirmation was done using ^1^H NMR (600 MHz, DMSO-d6): δ 9.97 (s, 2H), 8.89 (s, 4H), 8.80 (s, 4H), 8.21 (d, J = 6.3 Hz, 4H), 8.01 (d, J = 8.2 Hz, 4H), 7.86–7.79 (m, 6H), 7.21 (d, J = 8.2 Hz, 4H), −2.90 (s, 2H), and ESI-MS: m/z calculated for C_44_H_30_N_4_O_2_ [M + H]^+^: 647.2441.

### 2.4 Synthesis of TK-CPT

The thioketal (TK) (10.08 mg, 0.04 mmol), 4-dimethylaminopyridine (DMAP) (5.86 mg, 0.048 mmol), and 1-(3-dimethylaminopropyl)-3-ethylcarbodiimide hydrochloride (EDCI) (9.20 mg, 0.048 mmol) were combined and added to 5 mL of dichloromethane in a concise process and stirred in an ice bath for 20 min. Subsequently, dropwise, 3 mL of dichloromethane-dissolved CPT (6.97 mg, 0.02 mmol) was meticulously added and stirred for 24 h at room temperature. Post-reaction, the solution underwent vacuum concentration to yield the resultant mixture. This mixture underwent separation and purification via silica gel column chromatography utilizing dichloromethane:methanol gradient elution ranging from 70:1 to 50:1. Ultimately, the white product TK-CPT was obtained with a yield of 40%. TK-CPT was confirmed using ^1^H NMR and mass spectrometry (MS). ^1^H NMR (600 MHz, DMSO-d6): δ 8.70 (s, 1H), 8.13 (q, 2H), 7.87 (t, J = 7.6 Hz, 1H), 7.72 (t, J = 7.5 Hz, 1H), 7.24 (s, 1H), 5.50 (s, 2H), 5.32 (q, 2H), 2.94–2.76 (m, 4H), 2.69 (t, J = 7.2, 3.4 Hz, 2H), 2.38 (t, J = 7.0 Hz, 2H), 2.19–2.11 (m, 2H), 1.57 (s, 3H), 1.50 (s, 3H), 0.93 (t, J = 7.4 Hz, 3H). ESI-MS: m/z calculated for C_29_H_30_N_2_O_7_S_2_ [M + H]^+^: 583.1565.

### 2.5 Synthesis of CTT_2_

TK-CPT (11.65 mg, 0.02 mmol), DMAP (2.93 mg, 0.024 mmol), and EDCI (4.60 mg, 0.024 mmol) were included in 5 mL of dichloromethane and mixed in an ice bath for a duration of 20 min. After that, a solution of TPPOH_2_ (25.87 mg, 0.04 mmol) dissolved in 4 mL of dichloromethane was added once, and the amalgamation reacted at room temperature for 24 h. Subsequently, the solution was subjected to vacuum concentration to obtain the resulting mixture. The resulting combination was isolated and refined using a silica gel column chromatography method, employing a gradient elution of dichloromethane and methanol ranging from 100:1 to 70:1. Ultimately, the purple product CTT_2_ was obtained with a commendable yield of 86%. CTT_2_ was confirmed using ^1^H NMR and mass spectrometry (MS). ^1^H NMR (600 MHz, DMSO-d6): δ 9.98 (s, 1H), 8.90 (d, J = 12.0 Hz, 2H), 8.80 (d, J = 13.6 Hz, 6H), 8.38 (s, 1H), 8.25–8.19 (m, 6H), 8.06 (d, J = 8.5 Hz, 1H), 8.01 (d, J = 8.3 Hz, 2H), 7.89 (d, J = 8.0 Hz, 1H), 7.86–7.80 (m, 6H), 7.77 (t, 1H), 7.57 (t, J = 7.5 Hz, 1H), 7.49 (d, J = 8.3 Hz, 2H), 7.25 (s, 1H), 7.21 (d, J = 8.4 Hz, 2H), 5.50 (s, 2H), 5.07 (q, 2H), 3.01–2.87 (m, 8H), 2.15 (q, J = 7.3 Hz, 2H), 1.68 (s, 3H), 1.63 (s, 3H), 0.94 (t, J = 7.4 Hz, 3H), −2.96 (s, 2H). ESI-MS: m/z calculated for C_73_H_58_N_6_O_8_S_2_ [M + H]^+^: 1211.3829.

### 2.6 Synthesis of CTT_2_P

(3-Carboxypropyl) triphenyl phosphonium bromide (PPh_3_, 4.29 mg, 0.01 mmol), DMAP (1.47 mg, 0.012 mmol), and EDCI (2.30 mg, 0.012 mmol) were meticulously measured and introduced into 2 mL of dichloromethane and mixed in an ice bath for a duration of 20 min. Subsequently, 3 mL of CTT_2_ (12.11 mg, 0.01 mmol) dissolved in dichloromethane was carefully added to the reaction solution, and the amalgamation underwent stirring for 24 h at room temperature. Post-reaction, the solution underwent vacuum concentration to yield the resultant mixture. This resultant mixture was separated and purified using silica gel column chromatography, employing a gradient elution of dichloromethane: methanol within the range of 70:1 to 20:1. Ultimately, the purple-black product CTT_2_P was obtained, demonstrating a commendable yield of 66%. The structure of CTT_2_P was confirmed using ^1^H NMR and mass spectrometry (MS). ^1^H NMR (600 MHz, DMSO-d6): δ 8.93–8.71 (m, 8H), 8.38 (s, 1H), 8.27 (d, J = 8.2 Hz, 2H), 8.25–8.19 (m, 6H), 8.07 (d, J = 8.5 Hz, 1H), 7.97–7.89 (m, 10H), 7.87–7.81 (m, 12H), 7.78 (t, 1H), 7.62 (d, J = 8.3 Hz, 2H), 7.58 (t, J = 7.5 Hz, 1H), 7.50 (d, J = 8.3 Hz, 2H), 7.25 (s, 1H), 5.50 (s, 2H), 5.05 (q, 2H), 3.86–3.79 (m, 2H), 3.08–3.04 (m, 2H), 3.02–2.88 (m, 8H), 2.15 (q, J = 7.4 Hz, 2H), 2.09–2.00 (m, 2H), 1.68 (s, 3H), 1.63 (s, 3H), 0.94 (t, J = 7.4 Hz, 3H), −2.98 (s, 2H). ESI-MS: m/z calculated for C_95_H_78_N_6_O_9_S_2_P^+^Br^−^ [M-Br^-^]^+^: 1541.4982.

### 2.7 ROS response test of CTT_2_P

10 mg of CTT_2_P was dissolved in d6-DMSO and irradiated with a 280 mW cm^−2^, 660 nm laser for 1 min and 5 min, respectively. The ROS response of CTT_2_P was observed using ^1^H NMR.

### 2.8 Preparation of CTT_2_P@B NPs

CTT_2_P@B NPs were synthesized using a desolvation method. Specifically, 15 mg of Bovine Serum Albumin (BSA) was dissolved in 1 mL of deionized water. After stirring to achieve complete dissolution, the pH was accurately set to 9.0 by adding a 0.1 M NaOH solution. Afterwards, a solution of CTT_2_P dissolved in acetone, measuring 4 mL, was added to the aqueous phase at a steady rate of 1 mL** **min^−1^, while keeping the stirring rate at 500 r**·**min^-1^. After stirring for 30 min, 10 μL of 8% glutaraldehyde was incorporated, and the solution underwent a curing process for 12 h. Finally, the solution was centrifuged at 12,000 rpm for 20 min. The resulting precipitate was subjected to two additional resuspension and centrifugation with deionized water. The supernatant was retained, and the precipitate was re-dispersed in 2 mL of deionized water before being stored at 4°C.

### 2.9 Transmission electron microscopy observation and size distribution

The Zetasizer nanoZS (Malvern Instruments) was used to determine the particle size and zeta potential of the nanomedicine CTT_2_P@B NPs. Furthermore, the shape of CTT_2_P@B NPs was observed using the TEM (JEM-1400 microscope).

### 2.10 EE and DL of CTT_2_P@B NPs

UV-vis spectroscopy was used to measure the absorbance of the supernatant, which was retained after centrifugation, at 415 nm. From the standard curve of CTT_2_P, the calculation of CTT_2_P@B NPs’ encapsulation efficiency and drug loading was determined.

EE%=weight of drug in nanoparticles/weight of total drug×100%

DL%=weight of drug in nanoparticles/weight of nanoparticles×100%

### 2.11 Properties of spectra

The absorbance of TPPOH_2_, CTT_2_, CTT_2_P, and CTT_2_P@B in DMSO was measured using UV-vis spectroscopy. The fluorescence emission spectra of TPPOH_2_, CTT_2_, CTT_2_P, and CTT_2_P@B under 420 nm excitation were measured using a multifunctional microplate reader.

### 2.12 *In Vitro* drug release study

*In vitro* release assays were conducted employing a centrifugation-based methodology. CTT_2_P@B NPs were evenly distributed in 1.5 mL of PBS (pH 7.4 and 5.0) containing 10% ethanol. Subsequently, they were placed in a constant-temperature shaker and incubated at 37°C. The nanosuspension was subjected to centrifugation at 12,000 r**·**min^−1^ for 10 min at specific time intervals (0.25, 0.5, 1, 2, 4, 6, 8, 10, 12, 24, 36, and 48 h), and 1 mL of supernatant was collected and supplemented with 1 mL of fresh buffer. Quantifying the CTT_2_P drug within the supernatant was executed utilizing UV-vis spectroscopy, facilitating the computation of the release rate at each designated time point.

### 2.13 *In Vitro* drug singlet oxygen detection

Solutions of TPPOH_2_, CTT_2_, CTT_2_P, and CTT_2_P@B NPs, each with a concentration of 5 μM and containing DPBF, were prepared. The DPBF concentration was set at 150 μg mL^−1^. Subsequently, a 2 mL sample solution was placed in a 24-well plate. In order to commence the experiment, every specimen was exposed to light with a wavelength of 660 nm and an intensity of 280 mW cm^−2^ for a duration of 16 min. UV-vis spectroscopy at 420 nm was diligently recorded at 1-min intervals throughout the irradiation period. The manifestation of generated singlet oxygen was indicated by the discernible reduction in DPBF absorption at 420 nm.

### 2.14 Cell culture and cellular ROS detection during irradiation

The murine breast cancer cells (4T1) were procured from Icell Biotech Co., Ltd. (Shanghai, China). The 4T1 cells were cultivated in Dulbecco’s Modified Eagle’s Medium (DMEM) supplemented with 10% (v/v) fetal bovine serum (FBS) and 1% (v/v) penicillin/streptomycin. The cells were cultivated in a cell incubator maintained at a temperature of 37°C with 5% CO_2_ (v/v).

To conduct experiments, 4T1 cells were placed in 12-well plates with a density of 1.5 × 10^5^ cells per well. After incubating for 24 h, the 4T1 cells were treated with 5 μM CPT, TPPOH_2_, CTT_2_, CTT_2_P, and CTT_2_P@B NPs individually for a duration of 6 h. Afterwards, the cells were treated with 10 μM DCFH-DA for a duration of 20 min, followed by a PBS wash. Subsequently, the cells were subjected to laser irradiation at a wavelength of 660 nm and intensity of 280 mW** **cm^−2^ for a period of 1 min. The intracellular production of ROS was visualized using fluorescence microscopy.

### 2.15 Subcellular Co-localization experiment

The intracellular localization of different categories of drugs was examined using Confocal Laser Scanning Microscopy (CLSM). In summary, 4T1 cells were grown in confocal dishes with a cell density of 50,000 cells per well. After 24 h of incubation, the existing medium was removed, and TPPOH_2_, CTT_2_, CTT_2_P, and CTT_2_P@B NPs (with a photosensitizer concentration of 10 μM), diluted in fresh medium, were introduced. Afterwards, Mito-tracker Green (200 nM) was added to all samples and incubated together in fresh medium for 30 min to mark the mitochondria. In the end, the liquid was removed, cells were washed with PBS, and a small amount of new liquid was introduced to each well prior to being placed under CLSM for observation. The excitation wavelength and emission spectra for each fluorescence index were as follows: TPPOH_2_, CTT_2_, CTT_2_P, and CTT_2_P@B NPs were excited at 405 nm, with fluorescence emission spectra collected at 600–660 nm. Mito-Tracker Green was excited at 488 nm, and fluorescence emission spectra were collected from 510 to 540 nm.

### 2.16 Mitochondrial membrane potentials assay

Mitochondrial depolarization in 4T1 cells was assessed employing the JC-1 probe. Briefly, 4T1 cells were placed in 12-well plates with a concentration of 2 × 10^5^ cells per well. After being incubated at 37°C for 24 h, the cells were subjected to 5 μM of CPT, TPPOH_2_, CTT_2_, CTT_2_P, and CTT_2_P@B NPs for a duration of 6 h. Then, the cells were exposed to a 660 nm, 280 mW cm^−2^ laser for 1 min, followed by an additional 18-h incubation period. The control group cells were treated with medium or carbonyl cyanide m-chlorophenyl hydrazone (CCCP) to ascertain the status of the membrane potential whether it remained normal or dissipated. Following the incubation period, the cells were rinsed with PBS, trypsinized, and then gathered in centrifuge tubes to be centrifuged. Afterward, 500 μL of JC-1 working solution were introduced and left to incubate at a temperature of 37°C for a duration of 20 min. After centrifugation, the liquid portion was discarded, and the cells were washed and centrifuged once using JC-1 Assay Buffer. Finally, the cells were resuspended to create a cell suspension. Flow cytometry analysis was performed using 525 nm (FITC channel) and 585 nm (PE channel) bandpass filters, with excitation at 488 nm.

### 2.17 Cytotoxicity test

4T1 cells were seeded in 96-well plates at a concentration of 5,000 cells per well using 100 μL of culture medium and incubated for 24 h. Afterwards, the cells were treated with different solutions of CPT, TPPOH_2_, CTT_2_, CTT_2_P, and CTT_2_P@B NPs (drug loading concentrations of 0.5, 1, 2, 5, 10, and 20 μM). For dark toxicity assessment, cells were exposed to drugs for a period of 24 h while being protected from any light exposure. For the assessment of phototoxicity, the cells underwent exposure to a laser with a wavelength of 660 nm and power density of 280 mW**·**cm^−2^ for a duration of 1 min, following continuous culturing for 6 h. After the irradiation, the cells were further cultured for 18 h. Then, a combination of CCK-8 and DMEM medium was introduced and incubated with the cells for 2 h, and the absorbance at 450 nm was determined using a microplate reader.

### 2.18 Cell apoptosis assay

4T1 cells were seeded in 6-well dishes with a density of 5 × 10^5^ cells per well and incubated for 24 h. The cells were treated with CPT, TPPOH_2_, CTT_2_, CTT_2_P, and CTT_2_P@B NPs solutions (drug concentration of 5 μM) for 6 h. Following that, a laser with a wavelength of 660 nm and an intensity of 280 mW cm^−2^ was used for 1 min of irradiation. The culture was then allowed to continue for 18 h post-irradiation. After two washes with PBS, the plates were trypsinized without EDTA, and the cells were collected in centrifuge tubes for centrifugation. Binding Buffer was added after resuspending and washing the single-cell suspensions with PBS. After being treated with Annexin-V FITC and PI for a duration of 15 min, the cells underwent evaluation of apoptosis using a flow cytometer (BD Canto II).

### 2.19 Hemolysis assay

Blood was obtained from mice, and erythrocytes were separated by centrifugation. The erythrocytes were subsequently diluted by washing with PBS before use. The red blood cell suspension was mixed with 10 μM solutions of CPT, TPPOH_2_, CTT_2_, CTT_2_P, and CTT_2_P@B NPs in the experiments. After incubation at 37°C for 2 h, the samples were centrifuged to collect the supernatant, which was then subjected to UV-vis spectroscopy analysis at 540 nm. PBS and Triton X-100 served as the negative and positive controls, respectively. Hemolysis was calculated using the following formula:

$$\text{Hemolysis }\left(\%\right)=\left({\text{OD}}_{\text{sample}}‐{\text{OD}}_{\text{negative}}\right)\;/\;\left({\text{OD}}_{\text{positive}}‐{\text{OD}}_{\text{negative}}\right)\times 100\%$$

### 2.20 Animal and tumor models

All Balb/c mice (4 weeks old, female) were procured from Jinan Pengyue Experimental Animal Breeding Co., Ltd. All animal studies followed the guidelines and protocols approved by the Animal Care Agency of Binzhou Medical University. The 4T1 tumor-bearing mouse model was established by subcutaneously injecting a 4T1 cell suspension (1 × 10^6^ cells) into the right abdomen of the mice. Mice were utilized for biodistribution and NIR fluorescence imaging studies when the tumor volume reached 200 mm^3^. Antitumor therapy was conducted using mice that had a tumor volume of 100 mm^3^.

### 2.21 *In Vivo* fluorescence imaging

The 4T1 tumor-bearing mice had their skin shaved and were divided into three groups randomly (*n* = 3 in each group). The mice were intravenously injected with 200 μL of CTT_2_P and CTT_2_P@B NPs (both containing 100 μg CTT_2_P). Mice were anesthetized and imaged using the IVIS Spectrum (Perkin-Elmer, United States) at 2, 4, 8, 10, and 24 h after injection. The imaging involved an excitation wavelength of 640 nm and a fluorescence emission wavelength of 700 nm. After 24 h post-injection, the mice, as mentioned above, were euthanized, and major organs were collected and imaged.

### 2.22 *In Vivo* anti-tumor efficacy

In each group, the mice with 4T1 tumors were randomly divided into six groups (*n* = 5) and given NS or CPT, TPPOH_2_, CTT_2_, CTT_2_P, and CTT_2_P@B NPs intravenously. Each group was administered a single dose every other day, which is equal to a CPT dosage of 2 mg kg^−1^ Cel. After 10 h of injection, the tumors in the laser irradiation groups were subjected to a 660 nm laser for a duration of 1 min, with a power intensity of 280 mW** **cm^−2^. Subsequently, the therapeutic efficacy of each group was assessed by measuring tumor size every 2 days. The formula for calculating tumor volume is tumor volume (mm^3^) = (W^2^ × L)/2, where L denotes the greater diameter of the tumor and W denotes the lesser diameter of the tumor. After 12 days, the mice were euthanized and their organs (including the heart, liver, spleen, lung, and kidney) along with solid tumor tissues were removed for histological examination. The examination was conducted using the standard H&E staining method. TUNEL immunohistochemical stains were performed to analyze tumor tissue apoptosis.

### 2.23 Statistical analysis

The mean ± SD was used to express the quantitative data. The statistical analysis was performed utilizing the software GraphPad Prism 8.0. To compare two groups, the Student's t-test was used, while for comparing multiple groups, a one-way analysis of variance (ANOVA) was employed. Statistical significance was determined for *p*-values below 0. 05 (*p < 0. 05, **p < 0. 01, ***p < 0. 001, ****p < 0. 0001).

## 3 Results and discussion

### 3.1 Synthesis and characterization of prodrug nanoparticles

The molecular prodrug CTT_2_P is primarily synthesized through a three-step reaction (Supplementary Scheme S1). To begin with, TPPOH_2_ and the TK sensitive to ROS were created using the method described in the source. The structures of these compounds were validated through ^1^H NMR and ESI-MS analyses (Supplementary Figures S1−S3). Subsequently, CTT_2_, a novel ROS-cleavable compound, was synthesized by covalently coupling CPT with TPPOH_2_ using TK. Finally, the molecular prodrug CTT_2_P was synthesized from (3-carboxypropyl) triphenyl phosphonium bromide and CTT_2_ through an esterification reaction. The structures of the described compounds were confirmed through ^1^H NMR and ESI-MS analyses (Supplementary Figures S4−S9). We then measured the ROS response release for CTT_2_P. Supplementary Figure S10 illustrates a significant reduction in the NMR peak of the TK part of CTT_2_P after 1 min and 5 min of illumination, and a significant shift in the chemical shift of the CPT part of CTT_2_P. This indicates that there is enough ROS for the compound CTT_2_P to break TK bonds when exposed to a 280 mW cm ^2^, 660 nm laser for 5 min.

The prodrug, which is bound covalently, reduces the escape of the drug while it circulates throughout the body, guaranteeing a one-to-one ratio between the target and the administered drug *in vivo*. Due to its positive charge, the triphenylphosphine group facilitates targeting the mitochondria of cancer cells. Upon exposure to laser light, TK in CTT_2_P is cleaved, releasing CPT in response to the abundant ROS generated by the photosensitizer and those present in the mitochondria themselves. Hydrophobic CPT was selected as the chemotherapeutic drug not only for its anticancer activity and ease of modification but also for its ability to inhibit cellular respiration, leading to an excessive production of ROS in mitochondria (Sen et al., 2004). Conversely, the excessive ROS produced by photosensitizers and CPT can effectively induce mitochondrial apoptosis, triggering cell apoptosis.

**FIGURE 1:**
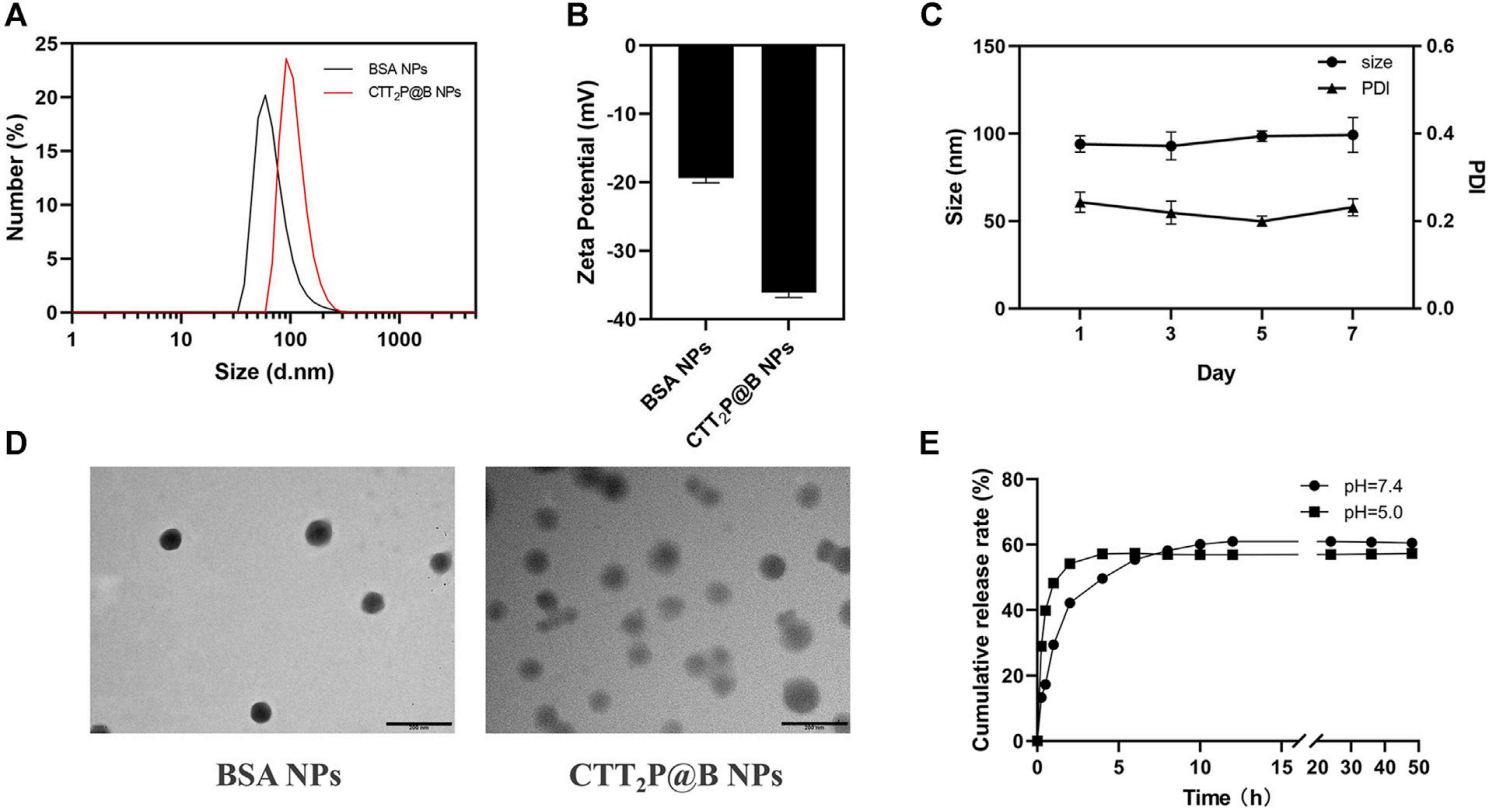
Analysis of CTT_2_P@B nanoparticles. **(A)** Particle size distribution of blank BSA NPs and CTT_2_P@B NPs. **(B)** Zeta potential of blank BSA NPs and CTT_2_P@B NPs. **(C)** Stability of CTT_2_P@B NPs in deionized aqueous solution. **(D)** TEM images of blank BSA NPs and CTT_2_P@B NPs. Scale bar = 200 nm. **(E)** The release of CTT_2_P from CTT_2_P@B NPs in a PBS solution at 37°C was examined using *in vitro* testing.

We employed desolvation to encapsulate CTT_2_P into BSA nanoparticles, yielding CTT_2_P@B NPs. To validate the successful synthesis of CTT_2_P@B, blank BSA NPs were synthesized as a control using the same method. The size and distribution of hydrated particles in blank BSA NPs and CTT_2_P@B NPs were first measured using dynamic light scattering (DLS) (Figures 1A,B). The results revealed that the hydrated particle sizes of blank BSA NPs and CTT_2_P@B NPs were 64.35 ± 5.09 nm and 92.89 ± 4.06 nm, respectively. Additionally, the potentials of blank BSA NPs and CTT_2_P@B NPs were −19.37 ± 0.70 mV and −36.13 ± 0.68 mV, respectively. The CTT_2_P@B NPs exhibited a size approximately 28 nm more prominent than the blank BSA NPs, and the potential had an absolute value of approximately 17 mV higher. These notable changes in particle size and potential indicated the successful encapsulation of CTT_2_P in BSA.

Furthermore, as indicated by prior research, the higher the magnitude of the potential, the increased stability of the nanoparticles was observed (Khan et al., 2016). This implies that CTT_2_P@B NPs exhibited enhanced stability compared to blank BSA NPs. Notably, the negatively charged surface of the NPs enhances their resistance to nonspecific protein adsorption in the bloodstream, ultimately prolonging their circulation time, as observed in other studies (Yuan et al., 2012). The results obtained from transmission electron microscopy (TEM) were consistent with the measured hydrated particle size using dynamic light scattering, confirming that CTT_2_P@B NPs are indeed more significant than their blank BSA counterparts (Figure 1D). All these indicators strongly suggest the successful encapsulation of CTT_2_P in BSA. TEM analysis revealed that CTT_2_P@B NPs exhibited a uniform distribution and good morphology, appearing as spherical particles. Subsequently, we evaluated the stability of CTT_2_P@B NPs in an aqueous solution. As shown in Figure 1C, The dimensions and polydispersity index (PDI) of CTT_2_P@B NPs remained stable over the course of 1 week, suggesting exceptional nanoparticle stability.

Utilizing the UV-vis spectroscopy of CTT_2_P, the standard curve was used to determine the encapsulation efficiency and drug loading of CTT_2_P@B NPs. The CTT_2_P@B NPs exhibited an encapsulation efficiency of 43.98% ± 2.38% and a drug loading of 2.75% ± 0.15%. Next, we measured the spectral properties of TPPOH_2_, CTT_2_, CTT_2_P, and CTT_2_P@B (Supplementary Figures S11, S12). As observed in the UV spectra, the maximum absorbance of TPPOH_2_, CTT_2_, and CTT_2_P around 420 nm gradually decreased with the successive modification of chemical bonds. However, due to BSA encapsulation, CTT_2_P@B exhibits a BSA UV absorption peak around 280 nm, and the maximum absorbance at 420 nm is smaller than that of CTT_2_P. Similarly, based on the fluorescence emission patterns, the maximum emission wavelengths of TPPOH_2_, CTT_2_, CTT_2_P, and CTT_2_P@B gradually decreased. Additionally, we examined the drug release pattern of CTT_2_P@B NPs in simulated physiological and tumor-acidic conditions. The drug release rate of CTT_2_P@B NPs in PBS solutions with pH values of 7.4 and 5.0 was evaluated by examining the cumulative release over time (Figure 1E), the results indicated a faster drug release under acidic conditions. This accelerated release is attributed to BSA’s self-degradation and erosion in acidic environments, aligning with our designed system. In the acidic environment of tumor cells, CTT_2_P@B NPs can more readily release the molecular drug CTT_2_P into tumor cells, facilitating the effective targeting of mitochondria and the intended therapeutic function.

### 3.2 *In Vitro* detection of ROS

**FIGURE 2:**
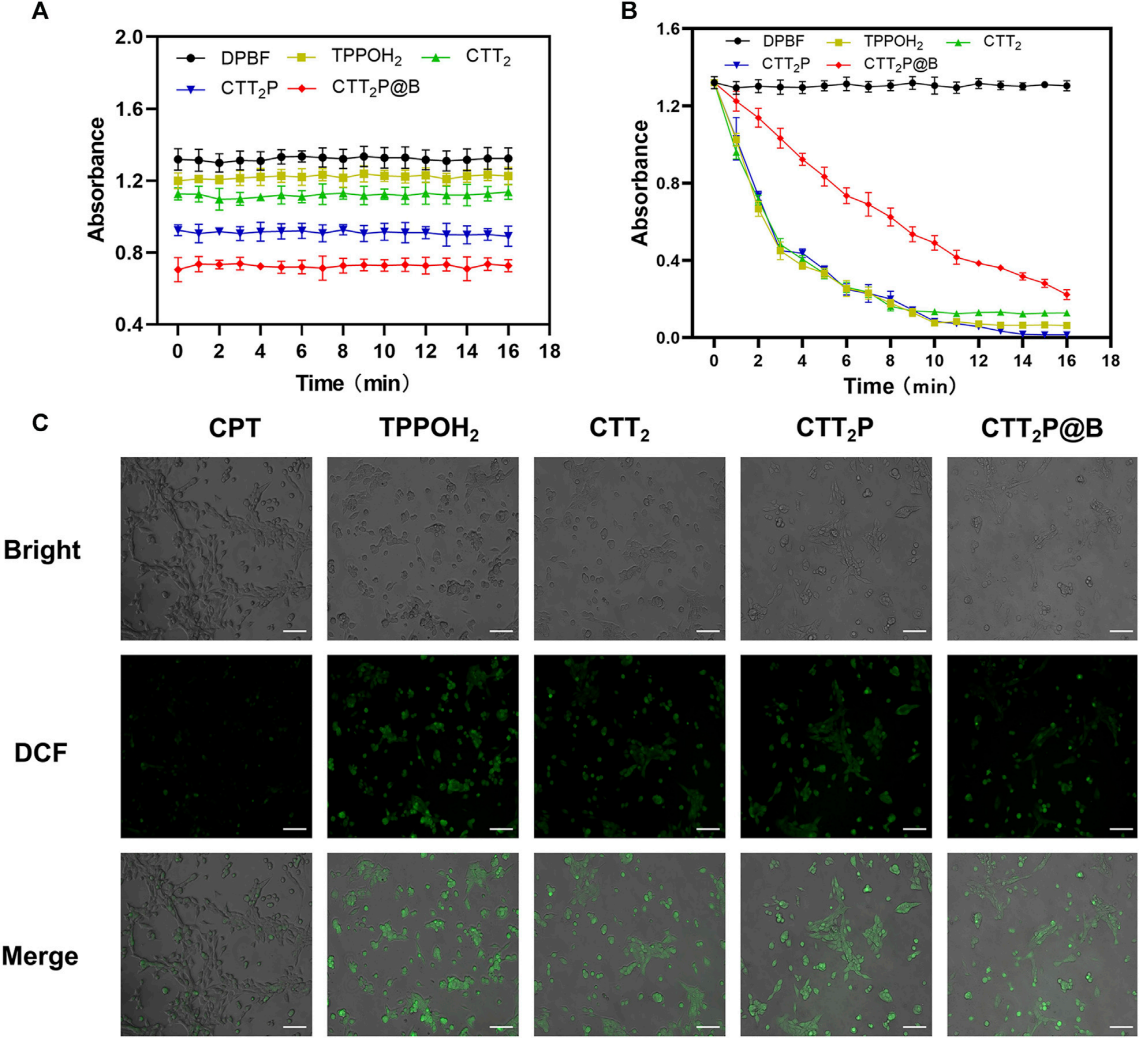
ROS generation detection *in vitro*. **(A)** The release of singlet oxygen from each drug was detected *in vitro* under dark conditions. Data were presented as the mean ± SD (*n* = 3). **(B)** Detection of singlet oxygen release of each drug under 660 nm laser irradiation at a power density of 280 mW** **cm^-2^ was performed *in vitro*. Data were presented as the mean ± SD (*n* = 3). **(C)** ROS generation was observed in 4T1 cells treated with each medication while being exposed to laser irradiation at a wavelength of 660 nm and intensity of 280 mW** **cm^−2^. Bright: Bright field. DCF: Green fluorescence represents intracellular ROS. Merge: Superimpose the image. Scale Bar = 100 μm.

1,3-Diphenylisobenzofuran (DPBF) can react with singlet oxygen (^1^O_2_) generated during the process. The absorption intensity of DPBF at 420 nm decreases with an increase in the concentration of ^1^O_2_ (Xu et al., 2018). Hence, DPBF functioned as a tool for assessing the ability of medications to produce singlet oxygen (^1^O_2_). Initially, we examined whether there was ^1^O_2_ release from the drug under dark conditions. In Figure 2A, based on the data, it is clear that the DPBF, TPPOH_2_, CTT_2_, CTT_2_P, and CTT_2_P@B NPs did not show any noticeable alterations in absorbance when kept in darkness. This suggests that the photosensitizer created did not generate ^1^O_2_ without light. We confirmed the release of ^1^O_2_ from the synthesized photosensitizer under laser irradiation by utilizing the absorbance change of the drug in the dark as a control. Figure 2B shows that the absorbance of DPBF also did not undergo significant changes under laser irradiation conditions, indicating that the presence of DPBF did not interfere with the experiment. The slope and degree of absorbance drop represent the rate of ^1^O_2_ production. The slopes of TPPOH_2_, CTT_2_, and CTT_2_P are nearly identical, suggesting a consistent rate of ^1^O_2_ release and an excellent ability to produce ^1^O_2_. However, the slope of absorbance decline for CTT_2_P@B NPs was slower than TPPOH_2_, CTT_2_, and CTT_2_P. The presence of nanoparticles leads to a decrease in the rate of ^1^O_2_ emission, resulting in a reduction of laser irradiation intensity.

Additionally, DCFH-DA staining was utilized to analyze the generation of intracellular ROS in 4T1 cells across all drug groups. DCFH-DA, an impermeable indicator of cellular permeability for reactive oxygen species (ROS), is hydrolyzed by esterases within cells and then oxidized by ROS to generate the fluorescent compound DCF. As observed through a fluorescence microscope (Figure 2C), the CPT group did not display noticeable fluorescence. In contrast, the TPPOH_2_, CTT_2_, CTT_2_P, and CTT_2_P@B NPs groups exhibited significant fluorescence, indicating that drugs modified with a photosensitizer still retained the ability to generate ROS upon irradiation in cells.

### 3.3 Evaluation of mitochondrial targeting function

**FIGURE 3:**
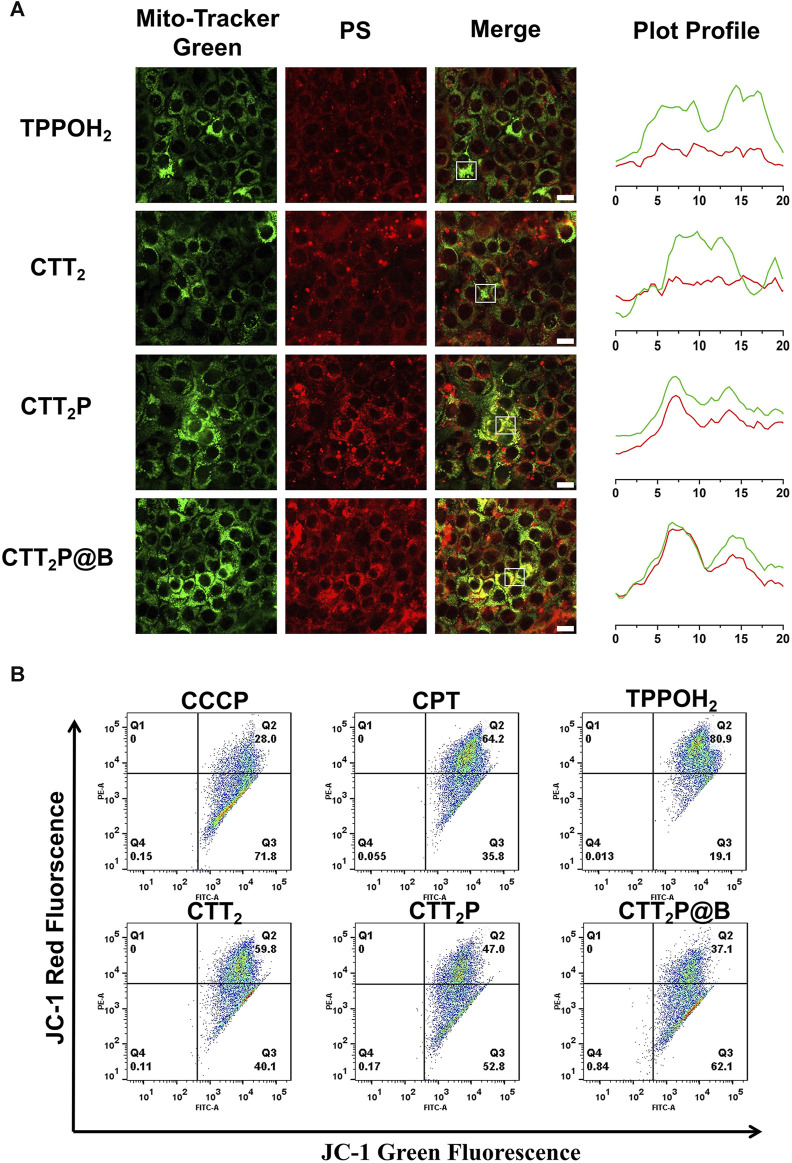
Evaluation of mitochondrial targeting function. **(A)** Confocal images of the mitochondrial sites of TPPOH_2_, CTT_2_, CTT_2_P, and CTT_2_P@B NPs in 4T1 cells. Mito-Tracker Green was used to stain the mitochondria in the green channel. The red channel was derived from the emission of the photosensitizer fraction (PS) itself. Merge stands for superimposed image. The Green and red curves in the Plot Profile represent the gray value of Mito-Tracker Green and PS, respectively. Scale bar = 20 μm. **(B)** Flow cytometry JC-1 method was used to analyze the mitochondrial function of cells treated with different drugs. Red fluorescence: normal mitochondria (J-aggregate); Green fluorescence: depolarized mitochondria (J-monomer).

To assess the drugs’ capacity to target mitochondria, the drugs were administered to 4T1 cells for a duration of 6 h, and the co-localization of the drug was analyzed using CLSM. Since our drugs were explicitly designed for mitochondrial targeting, we assessed mitochondrial localization (Figure 3A) through fluorescent double staining. We utilized the Mito-Tracker Green, a fluorescent probe for mitochondria, along with the red fluorescence emitted by our designed drugs. The yellow area in the overlapping images indicates the colocalization of the photosensitizer with Mito-Tracker Green. The results revealed that cells treated with CTT_2_P and CTT_2_P@B NPs exhibited clear colocalization with Mito-Tracker Green, in contrast to TPPOH_2_ and CTT_2_. However, the CTT_2_P@B NPs displayed a more pronounced yellow area than CTT_2_P alone, as the nanodrugs were more easily taken up by the cells, resulting in increased drug accumulation in the mitochondria. This observation aligns with our design concept; CTT_2_P and CTT_2_P@B NPs demonstrate effective mitochondrial localization, targeting the mitochondria upon entering tumor cells under the influence of triphenylphosphine groups.

Next, we evaluated drug-induced impairment of mitochondria in every group by examining the potential of mitochondrial membrane using the positively charged dye JC-1. Prior research has shown that the JC-1 probe is effective in identifying mitochondrial harm. In normal mitochondria, the green monomers of JC-1 can enter the cytoplasm and accumulate, forming numerous red J-aggregates. A change in fluorescence color from red to green suggests the presence of membrane potential decline and considerable damage to the mitochondria (Zhang et al., 2019). Therefore, we quantified mitochondrial damage in 4T1 cells induced by each drug using flow cytometry. The green fluorescence channel (FITC) represented “J-monomer,” while the red fluorescence channel (PE) represented “J-aggregate.” As illustrated in Figure 3B, after being exposed to laser, the CPT, TPPOH_2_, CTT_2_, CTT_2_P, and CTT_2_P@B NPs groups experienced a decrease in mitochondrial membrane potential (in the right lower quadrant) with loss rates of 35.8%, 19.1%, 40.1%, 52.8%, and 62.1%, respectively. The data indicate that CPT can induce moderate mitochondrial dysfunction, which is consistent with previous reports (Sen et al., 2004). Meanwhile, triphenylphosphine-modified CTT_2_P caused more damage to mitochondria than TPPOH_2_ and CTT_2_ because it more easily accumulated at the mitochondrial site. CTT_2_P@B NPs could enter cells more quickly and accumulate more drugs at the mitochondrial site, resulting in a higher rate of mitochondrial damage than the molecular drug CTT_2_P.

### 3.4 *In Vitro* anti-tumor studies

**FIGURE 4:**
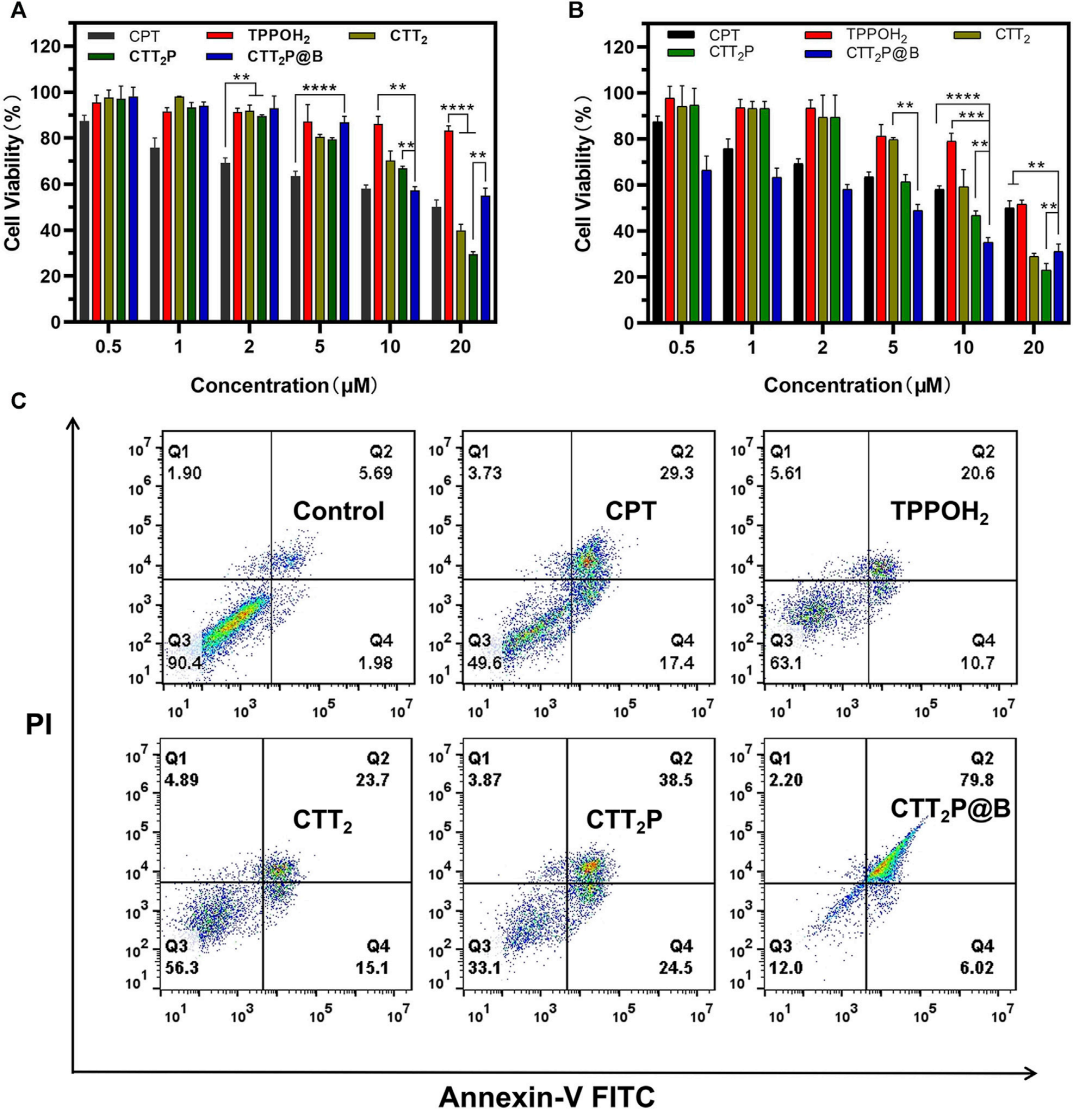
Evaluation of cell death *in vitro* through cytotoxicity assessment and examination of apoptosis and necrosis. **(A)** The *in vitro* cytotoxicity of 4T1 cells treated with CPT, TPPOH_2_, CTT_2_, CTT_2_P, and CTT_2_P@B NPs in the dark was assessed using the CCK-8 assay. Data were presented as the mean ± SD (*n* = 5). **p < 0.01, ****p < 0.0001. **(B)** The *in vitro* cytotoxicity of 4T1 cells treated with TPPOH_2_, CTT_2_, CTT_2_P and CTT_2_P@B NPs under laser irradiation (660 nm, 280 mW** **cm^−2^) was determined using the CCK-8 assay. Data were presented as the mean ± SD (*n* = 5). **p < 0.01, ****p < 0.0001. **(C)** Cell apoptosis and necrosis were analyzed using flow cytometry with Annexin V-FITC/PI double staining following treatment with various drugs at a concentration of 5 μM.

Mitochondria play a vital role in cellular respiration and serve as a significant generator of reactive oxygen species (ROS). Therefore, drugs targeting mitochondria can achieve maximum therapeutic efficacy. In order to examine the *in vitro* anticancer properties of the synthesized medications, we first evaluated the toxic effects of different drugs on 4T1 cells through the CCK-8 assay. 4T1 cells were treated with CPT, TPPOH_2_, CTT_2_, CTT_2_P, and CTT_2_P@B NPs in the dark or under laser irradiation (Figures 4A,B). Under dark conditions, TPPOH_2_, CTT_2_, CTT_2_P, and CTT_2_P@B nanoparticles exhibited more than 80% cell viability at concentrations below 10 μM, showing minimal harm to cells in contrast to CPT. CTT_2_ and CTT_2_P enhance the stability of the CPT lactone ring and reduce cytotoxicity by modifying the 20-hydroxyl group of CPT. When the concentration of CTT_2_, CTT_2_P, and CTT_2_P@B NPs was above 10 μM, CTT_2_, and CTT_2_P, as well as CTT_2_P@B NPs, showed obvious cytotoxicity in the dark, but CTT_2_P@B NPs showed less cytotoxicity than CTT_2_P. This indicates that high concentrations of CTT_2_, CTT_2_P, and CTT_2_P@B NPs still retain some cytotoxicity due to the presence of CPT under dark conditions. Simultaneously, CTT_2_P@B nanoparticles have the ability to decrease the cytotoxic effects of drugs by facilitating sustained release. When exposed to laser radiation (at a wavelength of 660 nm and intensity of 280 mW** **cm^−2^), TPPOH_2_, CTT_2_, CTT_2_P, and CTT_2_P@B NPs exhibited a significant reduction in cell viability compared to the CPT group. This indicates a dose-dependent and gradually escalating cytotoxic effect. This suggests that ROS-induced TK breakdown and CPT release enhance cell damage, further amplified by drugs targeting mitochondria. Similarly to the results obtained in the dark, the cytotoxicity of a high concentration (20 μM) of CTT_2_P@B NPs was reduced compared to CTT_2_P. This reduction was attributed to the relatively small amount of drug accumulation caused by the high concentration of CTT_2_P@B NPs.

Apoptosis rates, which include both early and late apoptosis, were examined through the use of the Annexin V-FITC/PI apoptosis detection kit and flow cytometry. As depicted in Figure 4C, laser-irradiated CTT_2_P@B NPs induced a significantly higher apoptosis rate (85.82%) compared to that of free drugs CTT_2_P (63%), CTT_2_ (38.8%), TPPOH_2_ (31.3%), and CPT (46.7%) under the same conditions. This result aligns with the trend observed in the *in vitro* cytotoxicity results and indicates that the cytotoxicity of the drugs is associated with both early and late apoptosis. Furthermore, it confirms that the prodrug CTT_2_P, modified with a mitochondrial targeting group, exhibits a more effective anti-tumor effect after being encapsulated with BSA.

### 3.5 *In Vivo* biodistribution

**FIGURE 5:**
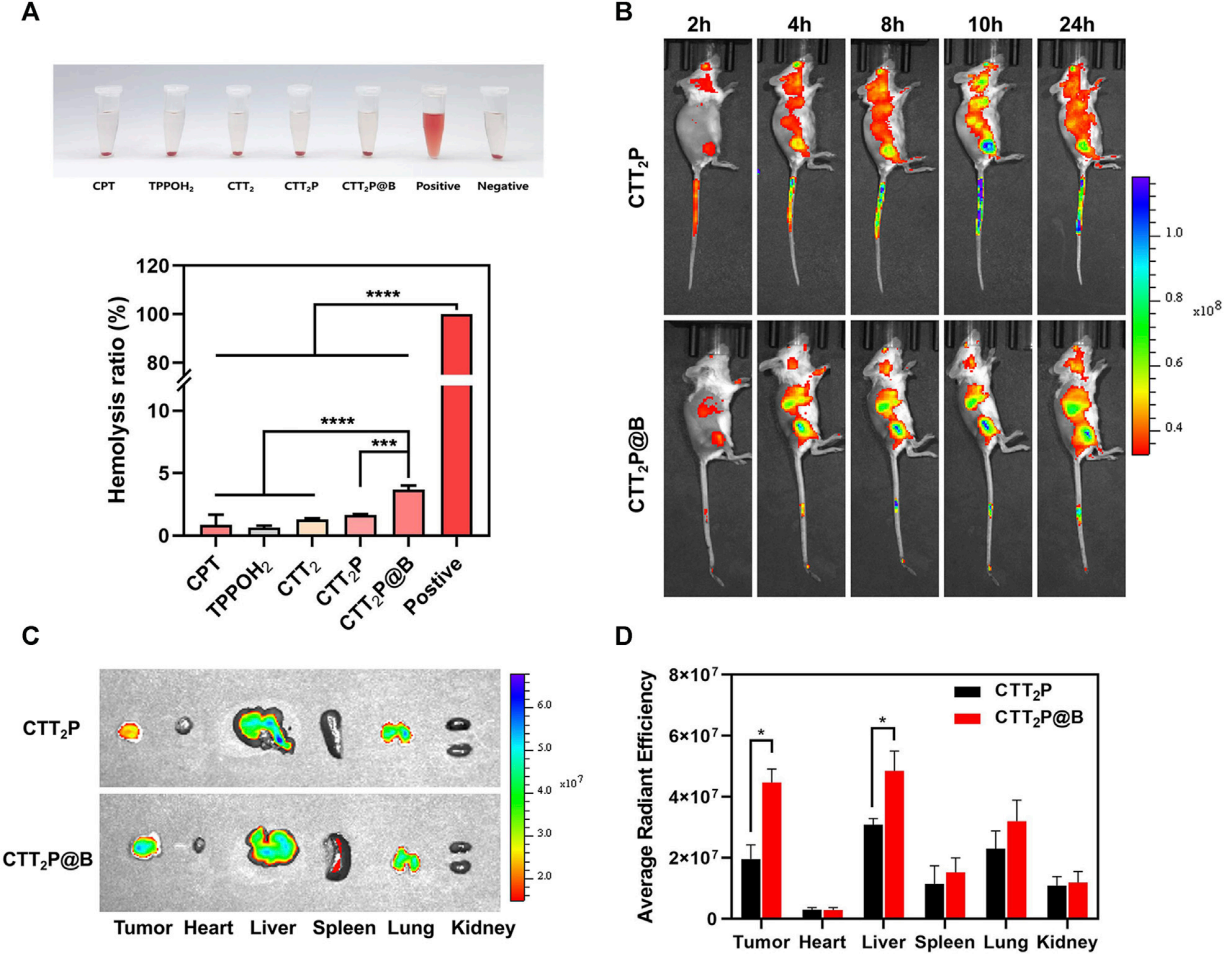
Biodistribution *in vivo*. **(A)** Blood compatibility test of CPT, TPPOH_2_, CTT_2_, CTT_2_P, CTT_2_P@B NPs. Data were presented as the mean ± SD (*n* = 3). ***p < 0.001, ****p < 0.0001. **(B)** Time-lapse live fluorescence imaging of mice with 4T1 tumors following the administration of free CTT_2_P and CTT_2_P@B NPs via intravenous injection. **(C)** Fluorescent images of major organs and tumors were obtained 24 h after injection, using *ex vivo* methods. **(D)** The mean fluorescence intensity of each organ and tumor was used to determine the biodistribution of free CTT_2_P and CTT_2_P@B NPs in mice. Data were presented as the mean ± SD (*n* = 3). *p < 0.05.

First, the hemolysis test verified the blood compatibility of each drug. As depicted in Figure 5A, the hemolysis rate of CPT, TPPOH_2_, CTT_2_, CTT_2_P, and CTT_2_P@B NPs at high concentrations did not exceed 5%. This indicates that the drugs exhibit good blood compatibility and can be safely administered via tail vein injection. We then investigated the biodistribution and drug delivery of CTT_2_P and CTT_2_P@B NPs in 4T1 tumor-bearing mice. CTT_2_P, possessing near-infrared fluorescence, obviates the need for physical packaging with other dyes, allowing it to integrate diagnosis and therapy. To monitor the distribution of medication in the body, an *in vivo* imaging system (IVIS) was utilized for fluorescence imaging. Images were captured at specific time intervals. As illustrated in Figure 5B and Supplementary Figure S13, higher accumulation was observed at the tumor site of mice treated with free CTT_2_P at 10 h. However, no significant accumulation occurred at 24 h, indicating rapid clearance of free CTT_2_P in mice.

On the other hand, CTT_2_P@B NPs showed notable buildup after 4 h, which lasted until 24 h. The fluorescence intensity at the tumor location reached its peak approximately 10 h post-injection, suggesting the highest accumulation of CTT_2_P@B NPs. Furthermore, *ex vivo* analysis of major organs and tumors was conducted 24 h post-injection to determine their distribution. As demonstrated in Figures 5C,D, in the heart and kidney of both groups, there were no apparent fluorescence signals, suggesting that neither drug group showed any signs of cardiotoxicity or nephrotoxicity. Liver and tumor tissues exhibited a notably elevated signal in the CTT_2_P@B NPs group compared to the free CTT_2_P group. The enhanced fluorescence signal in the entire circulatory system and *ex vivo* tumor tissues at the exact CTT_2_P dosage supports the long-circulating effect and tumor-targeting capabilities of CTT_2_P@B NPs.

### 3.6 *In Vivo* anti-tumor studies

**FIGURE 6:**
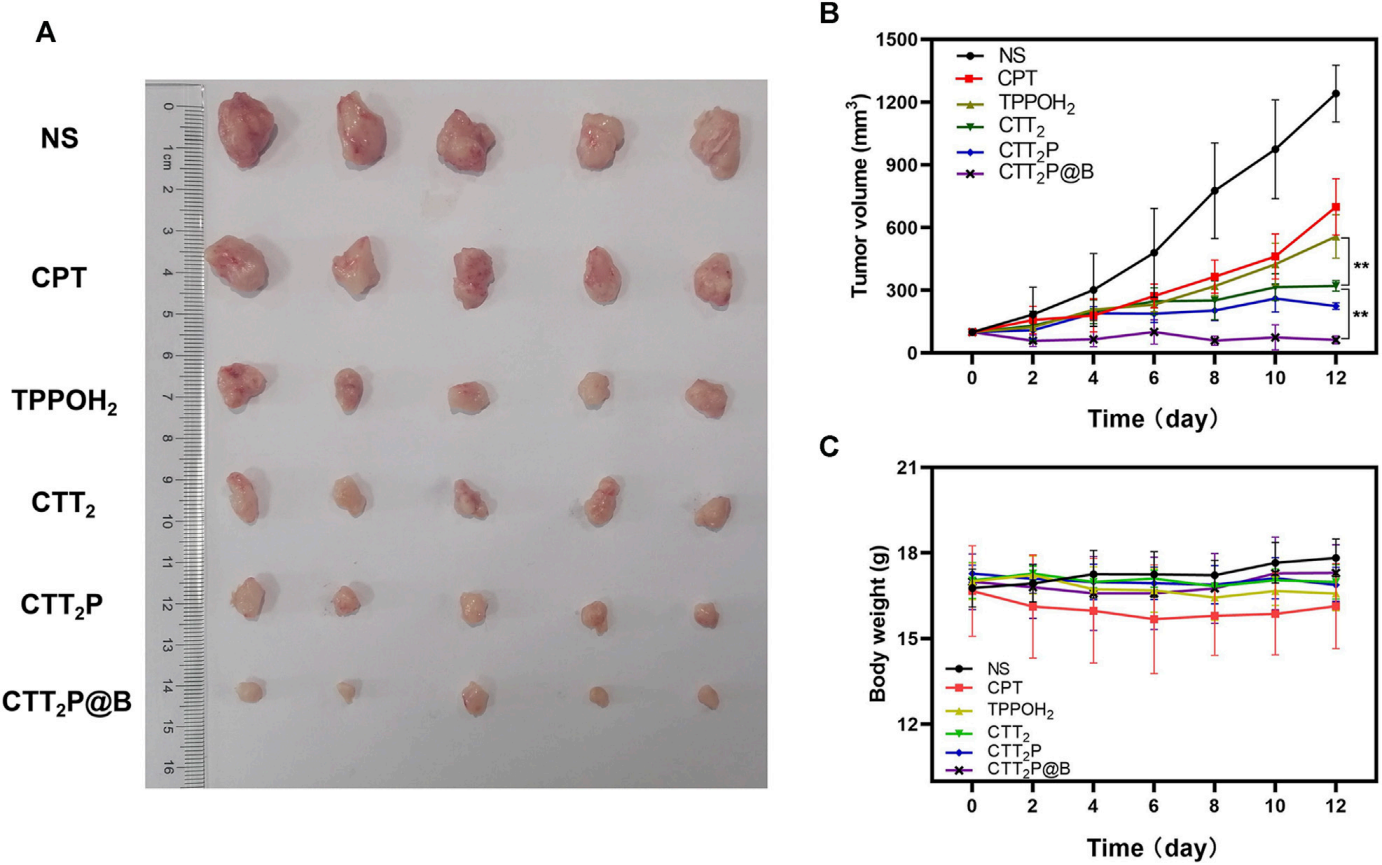
*In vivo* anti-tumor study of each drug in 4T1 tumor-bearing mice. **(A)** Tumor images of different drug administration treatments after the antitumor study. **(B)** During the administration, the growth of tumors in mice was observed in each group receiving treatment. Data were presented as the mean ± SD (*n* = 5), **p < 0.01. **(C)** The weight of mice in each treatment group was monitored throughout the administration period. Data were presented as the mean ± SD (*n* = 5).

The therapeutic efficacy of various drug groups on 4T1-bearing mice was validated through tail vein injection of CPT at a dosage equivalent to 2 mg** **kg^−1^. Based on the *in vivo* distribution results, laser irradiation was conducted 10 h after drug injection. Tumor images and tumor volume curves are depicted in Figures 6A,B. Tumor volume increased rapidly in mice treated with NS after laser irradiation, indicating an inability to impede tumor progression. The tumor volumes in the CPT and TPPOH_2_ groups were comparable. However, the anti-tumor effect was inadequate, suggesting that neither chemotherapy nor photodynamic therapy alone could inhibit tumor growth. The CTT_2_ group exhibited a more pronounced anti-tumor effect than the CPT and TPPOH_2_ groups. However, it still fell short of the CTT_2_P group, indicating that CTT_2_P, a molecular drug targeting the mitochondrial region, may produce a more potent cascade effect and a more significant therapeutic impact due to its proximity to the target. Due to enhanced blood circulation time and improved accumulation at the tumor site, the mice in the CTT_2_P@B group exhibited the highest level of tumor inhibition and anti-tumor impact. The *in vivo* tumor-suppressing properties of TPPOH_2_, CTT_2_, CTT_2_P, and CTT_2_P@B NPs were enhanced when exposed to laser radiation, in line with the findings from *in vitro* tests on cell toxicity and programmed cell death. This further validates that the molecular drug CTT_2_P, modified with a mitochondrial targeting group, exhibits superior therapeutic efficacy after encapsulation in BSA.

**FIGURE 7:**
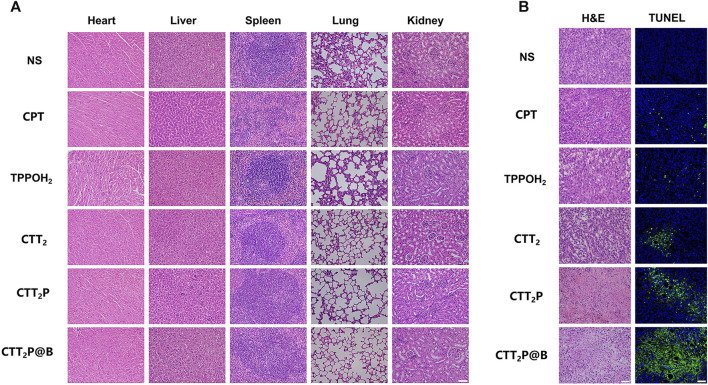
Investigation of the effects of each medication on mice with 4T1 tumors through pathological examination. **(A)** After administering various medications, the major organs (including the heart, liver, spleen, lung, and kidney) were subjected to H&E staining. Scale bar = 50 μm. **(B)** After administering various medications, tumors were subjected to H&E staining and TUNEL staining. Scale bar = 50 μm.

Furthermore, the mice’s body weight and major organ tissue sections were analyzed using hematoxylin-eosin (H&E) staining to evaluate the biological safety of each treatment group. As depicted in Figure 6C, during the entire experimental period, there was no significant decrease in the weight of mice in any of the groups. The findings suggest that the medications in every category did not have any significant effect on the wellbeing of the mice. No histomorphological changes, bleeding, or infiltration of inflammatory cells were observed in the heart, liver, spleen, lung, and kidney sections stained with H&E in all groups (Figure 7A). This suggests that the current dosage of these medications is biologically safe.

The tumor tissues were evaluated for histopathological features using H&E staining (Figure 7B). Compared with the NS group, the tumor cells in each treatment group exhibited cell atrophy and nuclear pyknosis. The CTT_2_P and CTT_2_P@B NPs groups demonstrated more pronounced nuclear pyknotic phenomena than the CPT, TPPOH_2_, and CTT_2_ groups. The apoptosis of tumor tissues was detected by terminal deoxynucleotidyl transferase-mediated dUTP-biotin nick end labeling (TUNEL) staining (Figure 7B), where blue represents living cells, and green represents apoptotic cells. The findings were consistent with the results of H&E staining. The absence of a notable disparity in cell apoptosis between the CPT group and the TPPOH_2_ group suggests that the anti-tumor effect of single chemotherapy and photodynamic therapy is suboptimal. On the other hand, the TPPOH_2_, CTT_2_, CTT_2_P, and CTT_2_P@B NPs groups showed a gradual increase in apoptosis, leading to a significant rise in the count of cells undergoing apoptosis. This further validates the excellent anti-tumor effect of CTT_2_P@B NPs.

## 4 Conclusion

In summary, we have successfully designed and synthesized a ROS-responsive prodrug, CTT_2_P, demonstrating high efficacy in inducing mitochondrial damage and subsequent apoptosis. Furthermore, we have formulated CTT_2_P@B NPs, encapsulating the prodrug CTT_2_P within BSA for efficient delivery and controlled release. The photosensitizer is covalently linked to CPT through a ROS-sensitive bond, TK. Additionally, the mitochondrial targeting group, triphenylphosphine, is incorporated to create the covalently linked prodrug, CTT_2_P. This covalent linkage enhances the specificity of chemotherapeutic drugs, minimizing side effects and synchronizing their *in vivo* distribution. The CTT_2_P@B NPs, formed by encapsulating the molecular prodrug CTT_2_P with BSA, facilitate the transport and release of the drug to tumor cells. Guided by the triphenylphosphine group, the molecular prodrug CTT_2_P selectively targets the mitochondrial site in tumor cells. Upon 660 nm laser irradiation, the photosensitizer component of the CTT_2_P prodrug generates a significant amount of ROS. These ROS induce mitochondrial damage and cleave the ROS-sensitive group TK in the prodrug. Furthermore, the accumulated CPT in the mitochondrial region exerts its chemotherapeutic action, generating additional ROS that disrupt the respiratory chain and promote mitochondrial apoptosis. Ultimately, through the synergistic effects of photodynamic therapy using a photosensitizer and chemotherapy with CPT, these drugs effectively enhance mitochondrial apoptosis, leading to cell apoptosis. The CTT_2_P@B molecular prodrug delivery system demonstrates low toxicity and a remarkable ability to induce mitochondrial apoptosis under laser irradiation. The findings of this research offer valuable information on the potential of organelle-specific methods to trigger apoptosis in cancerous cells.

## Data Availability

The original contributions presented in the study are included in the article/Supplementary Material, further inquiries can be directed to the corresponding authors.
