# Supplementary material for: Combining photodynamic therapy and cascade chemotherapy for enhanced tumor cytotoxicity: the role of CTT2P@B nanoparticles
Source: Front Bioeng Biotechnol. 2024 Feb 12;12:1361966. doi: 10.3389/fbioe.2024.1361966 (PMC10895035; doi:10.3389/fbioe.2024.1361966)
Supplement: Supplementary file 1 [file DataSheet1.docx]

Supplementary Material

Combining Photodynamic Therapy and Cascade Chemotherapy for Enhanced Tumor Cytotoxicity: The Role of CTT_2_P@B Nanoparticles





**Supplementary Scheme 1.** Flow chart for the design of CTT_2_P molecular prodrug synthesis.


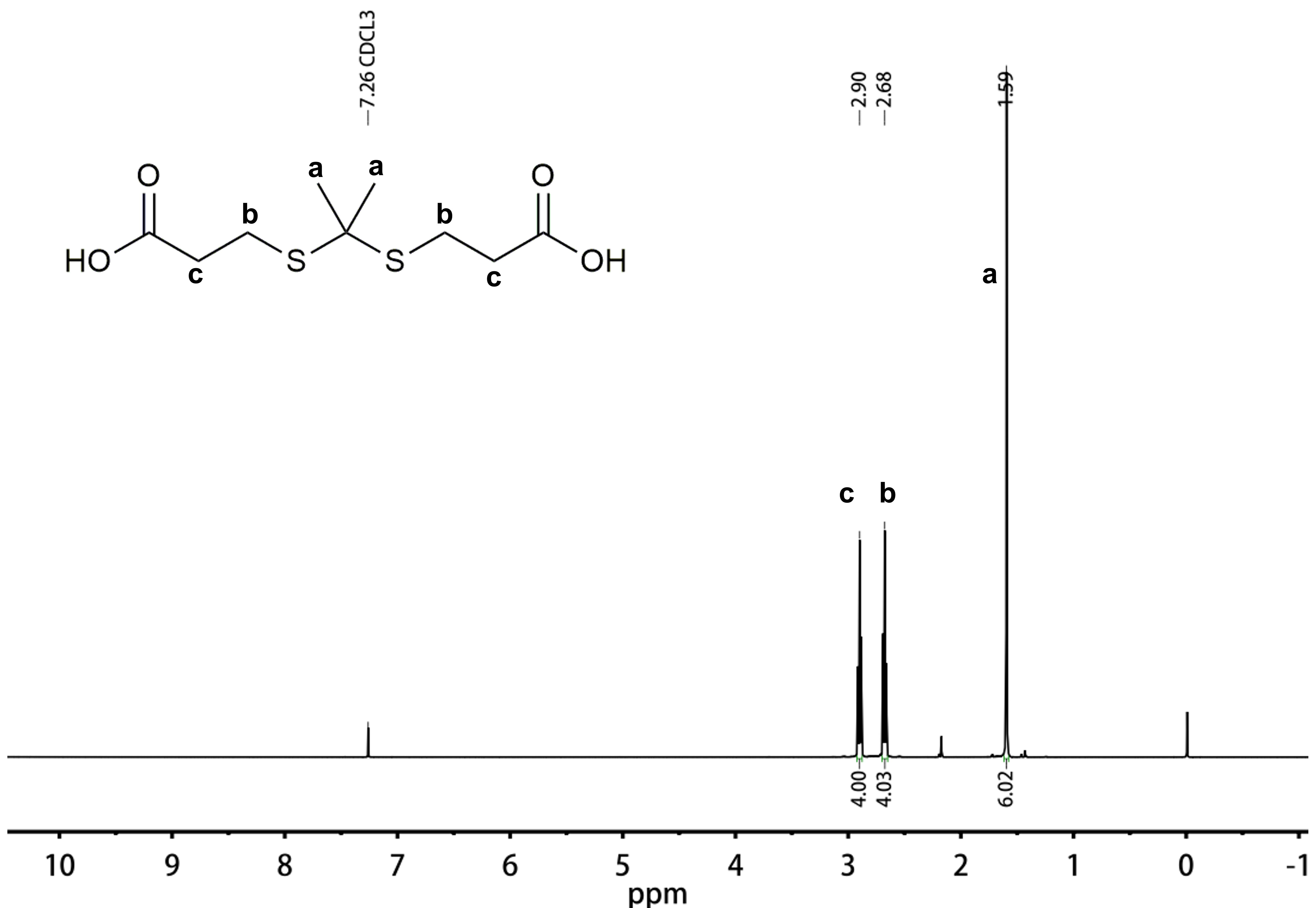


**Supplementary Figure 1.** ^1^H NMR of thioketal (TK).


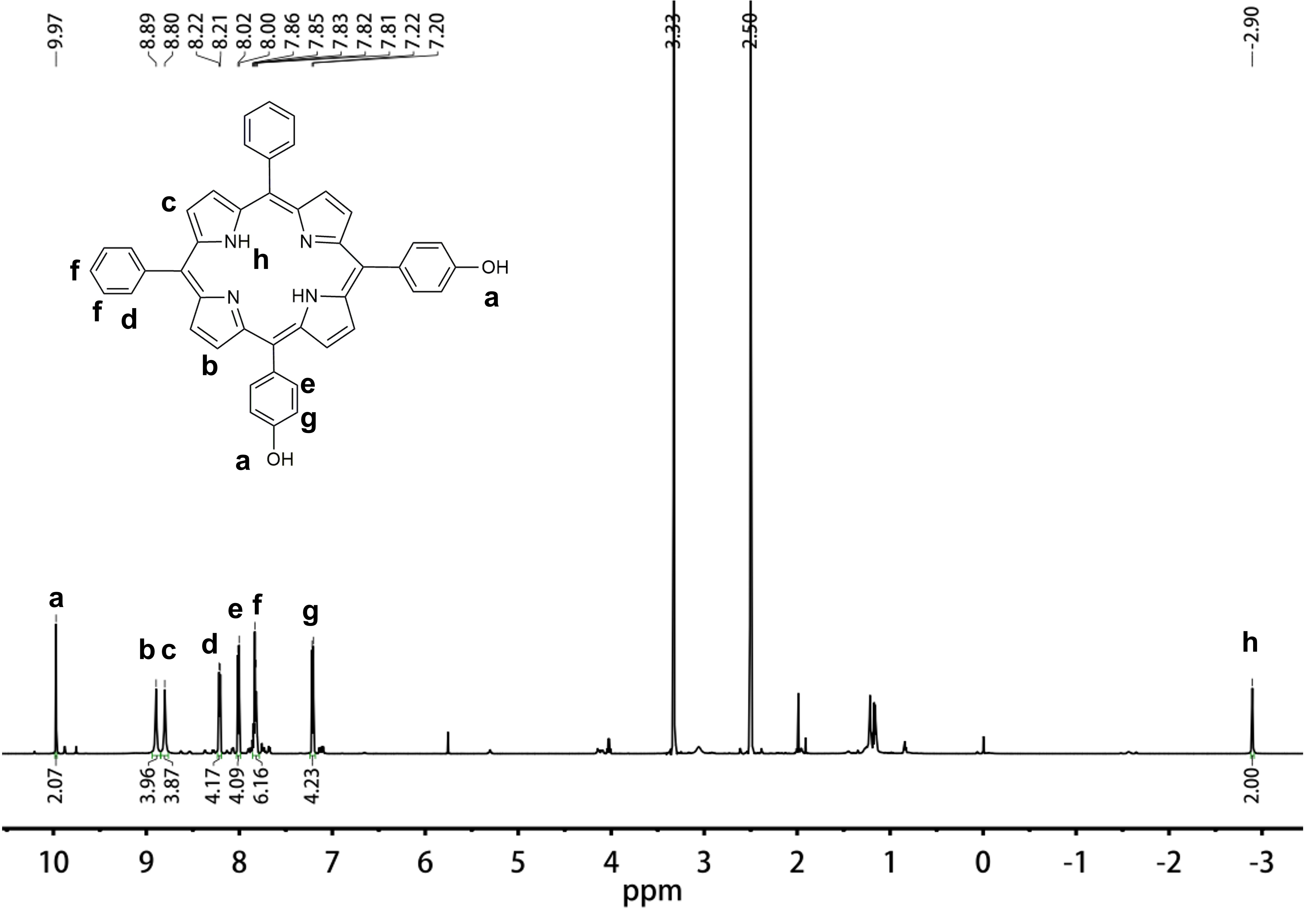


**Supplementary Figure 2.** ^1^H NMR of TPPOH_2_.


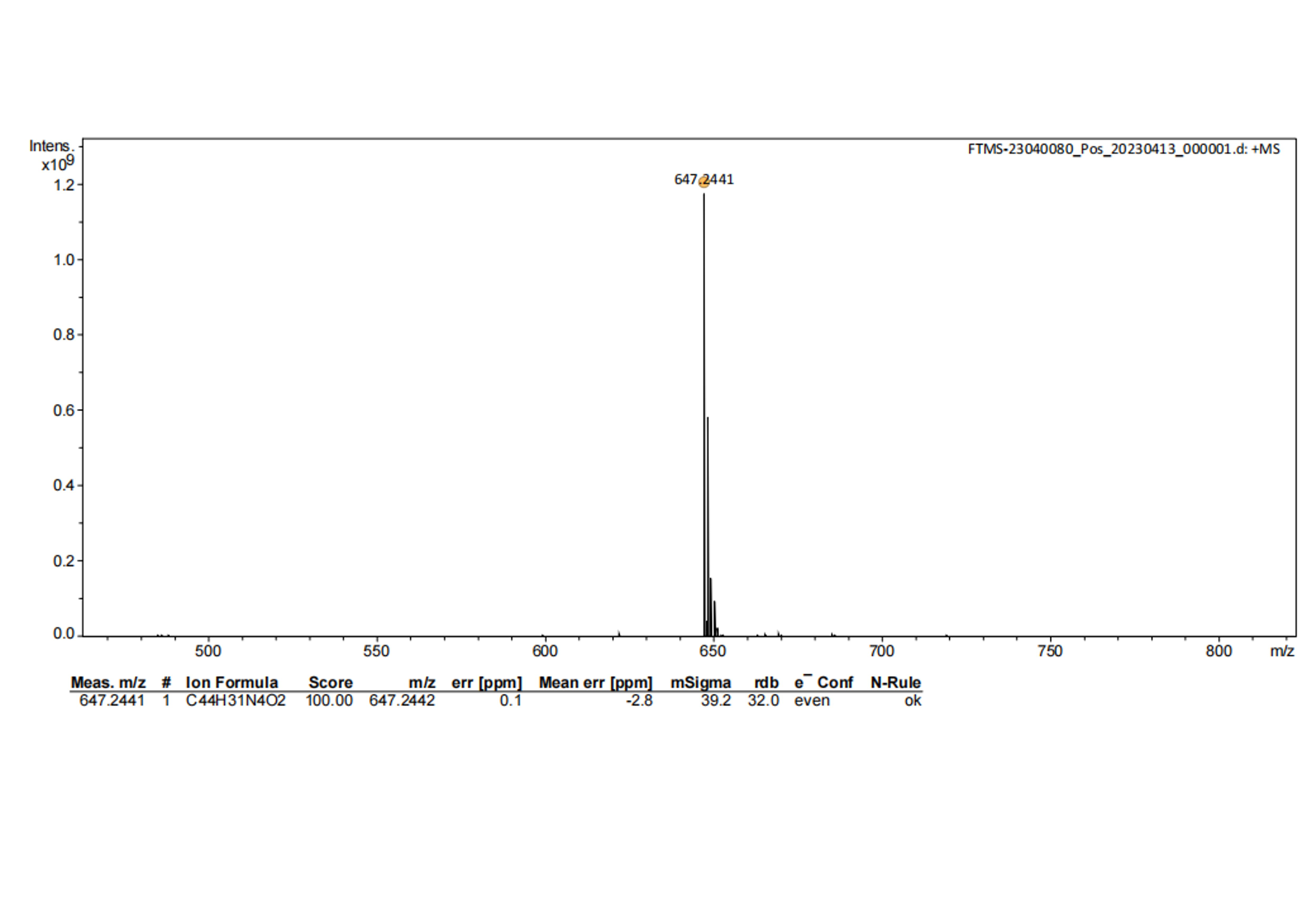


**Supplementary Figure 3.** Mass spectrum of TPPOH_2_: [M+H]^+^.


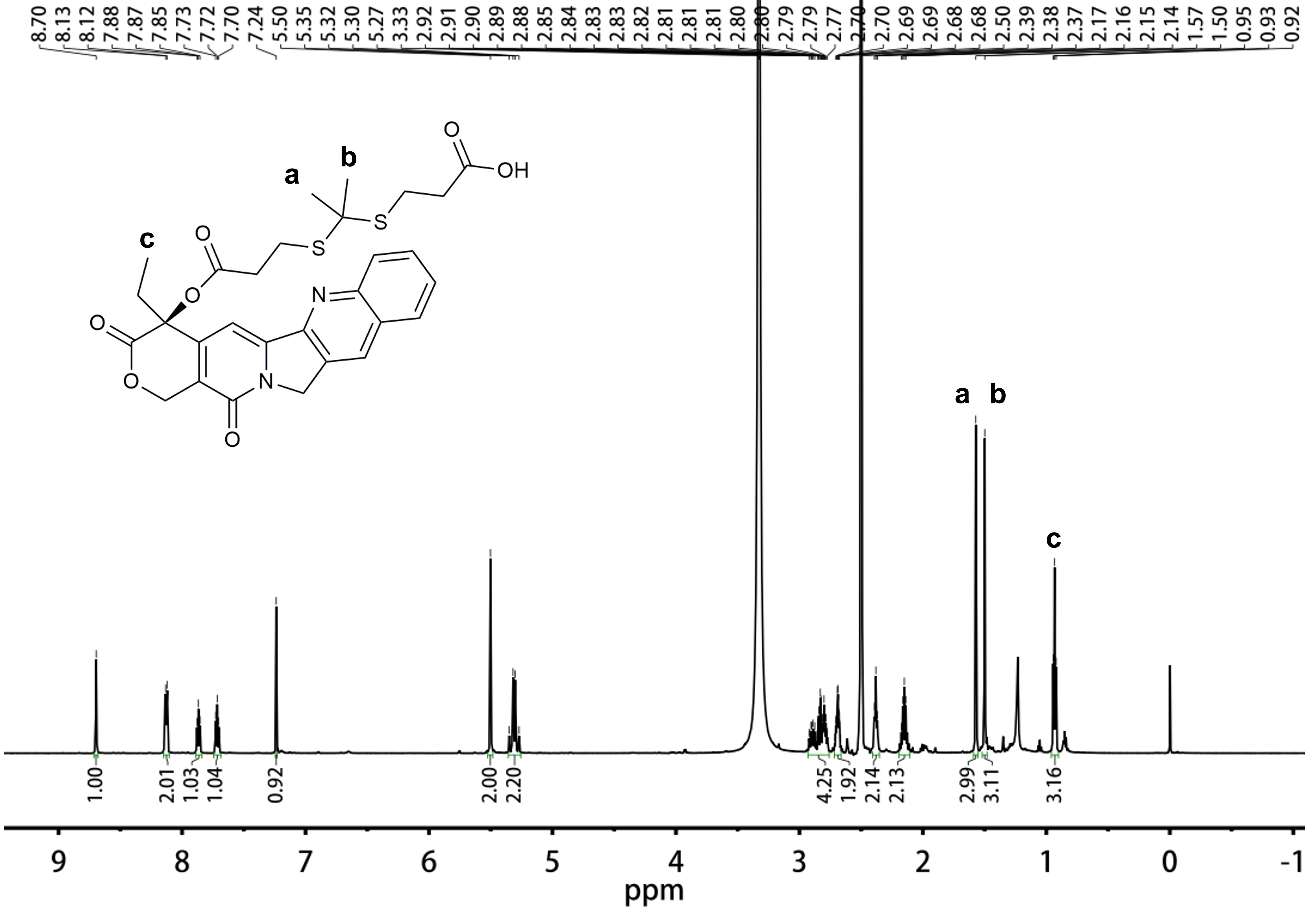


**Supplementary Figure 4.** ^1^H NMR of TK-CPT.


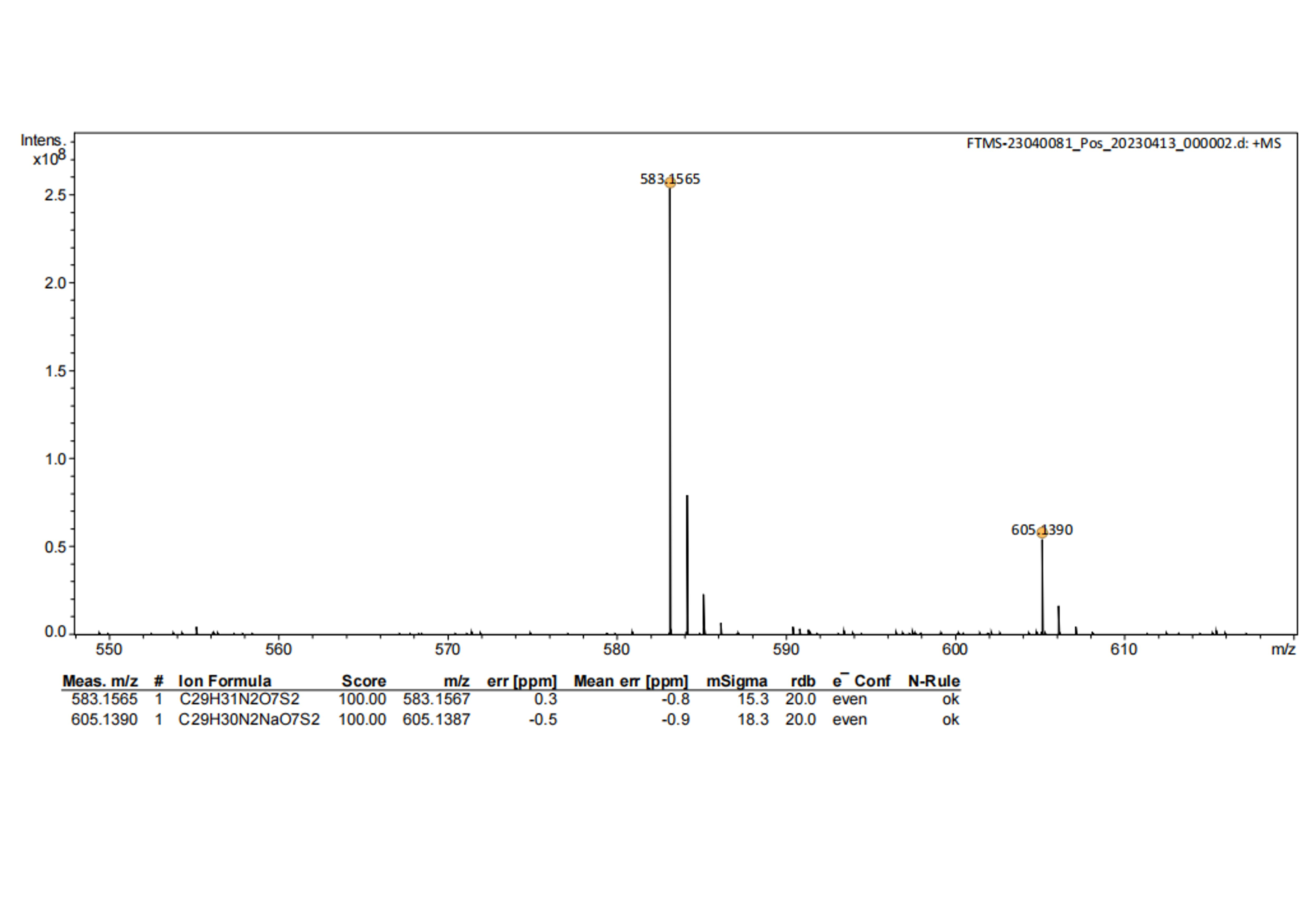


**Supplementary Figure 5.** Mass spectrum of TK-CPT: [M+H]^+^.


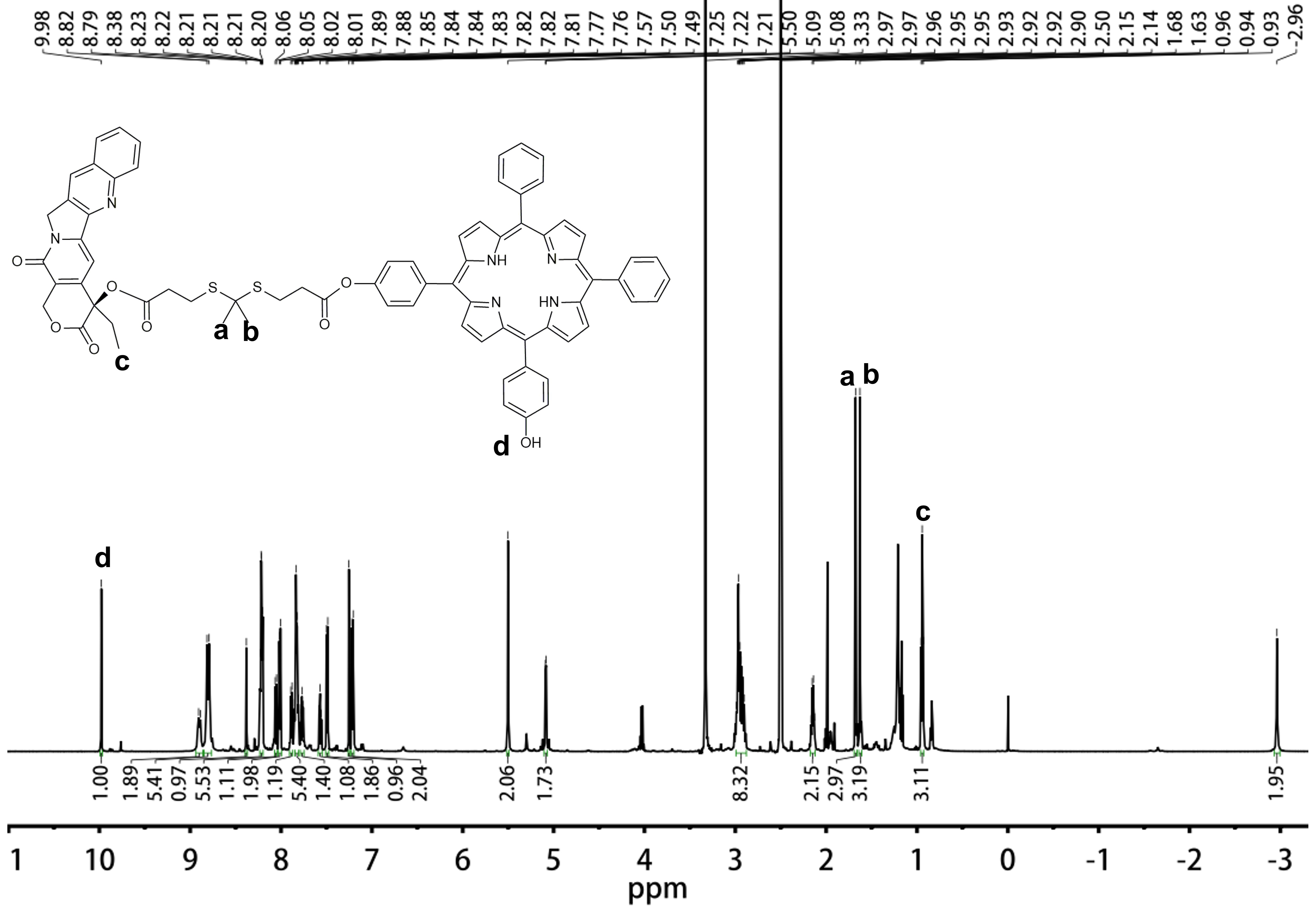


**Supplementary Figure 6.** ^1^H NMR of CTT_2_.


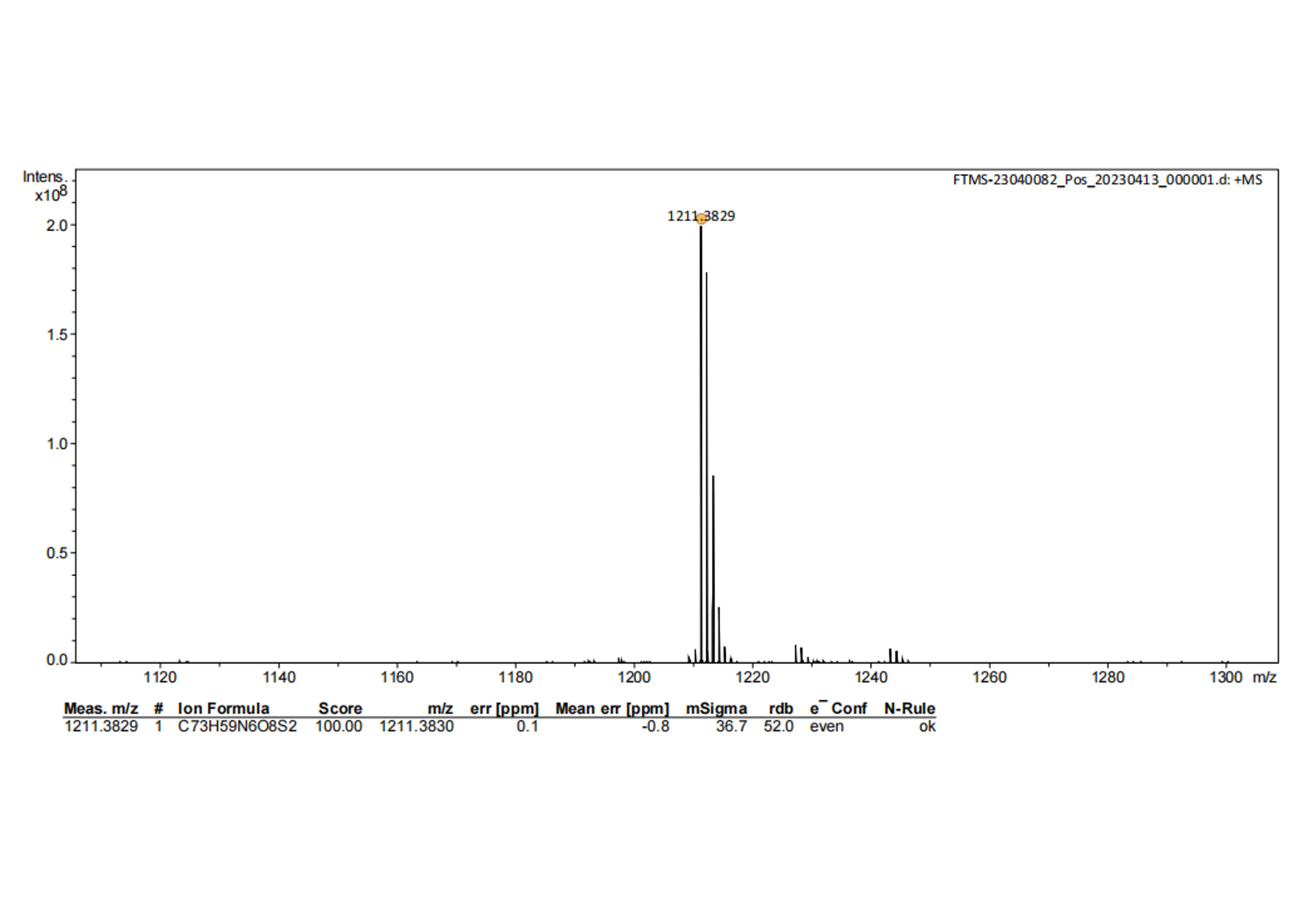


**Supplementary Figure 7.** Mass spectrum of CTT_2_: [M+H]^+^.


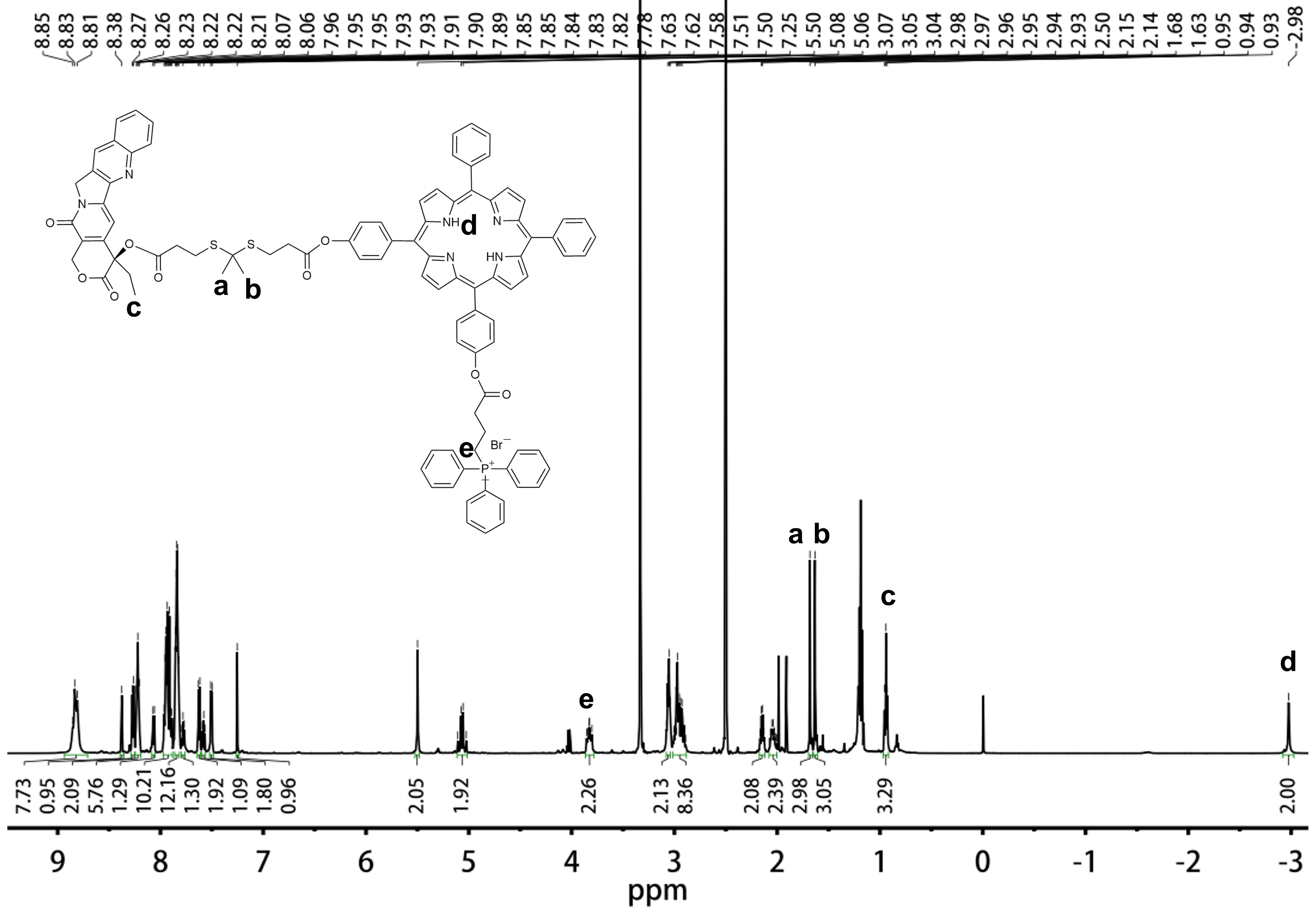


**Supplementary Figure 8.** ^1^H NMR of CTT_2_P.


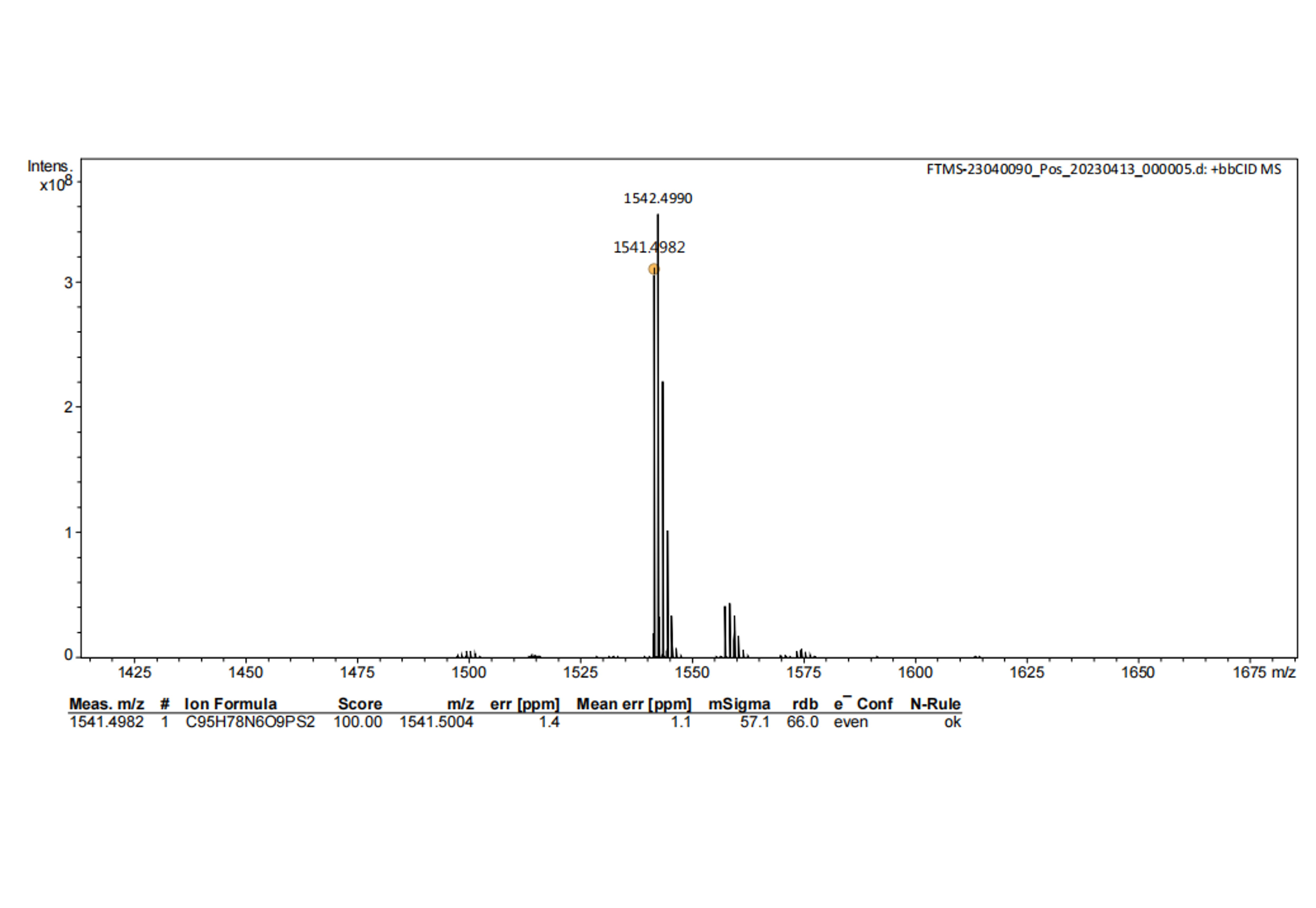


**Supplementary Figure 9.** Mass spectrum of CTT_2_P: [M-Br^-^]^+^.


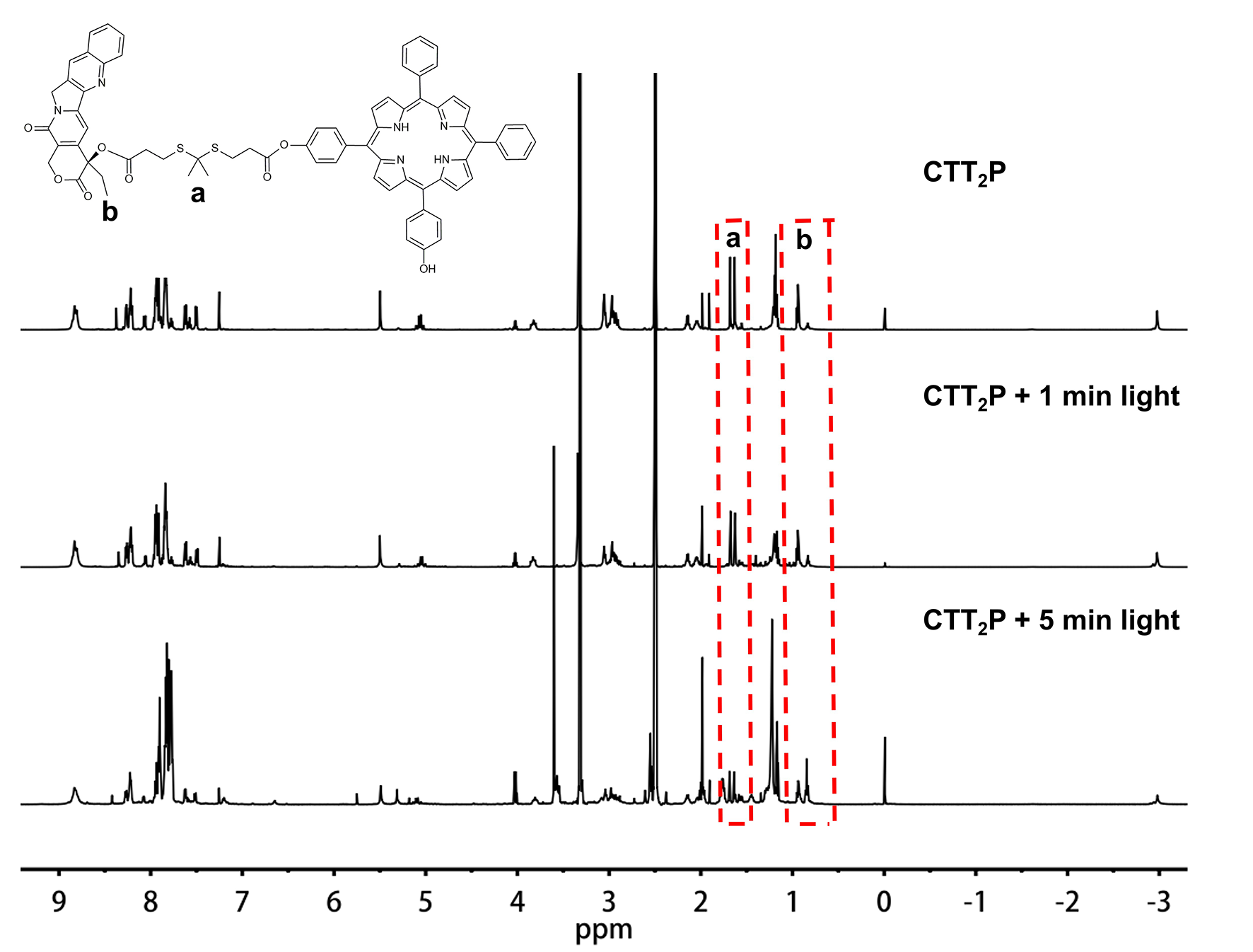


**Supplementary Figure 10.** The ROS response test of CTT_2_P and the chemical shift changes of CPT under laser irradiation at 660 nm, 280 mW·cm^-2^ .


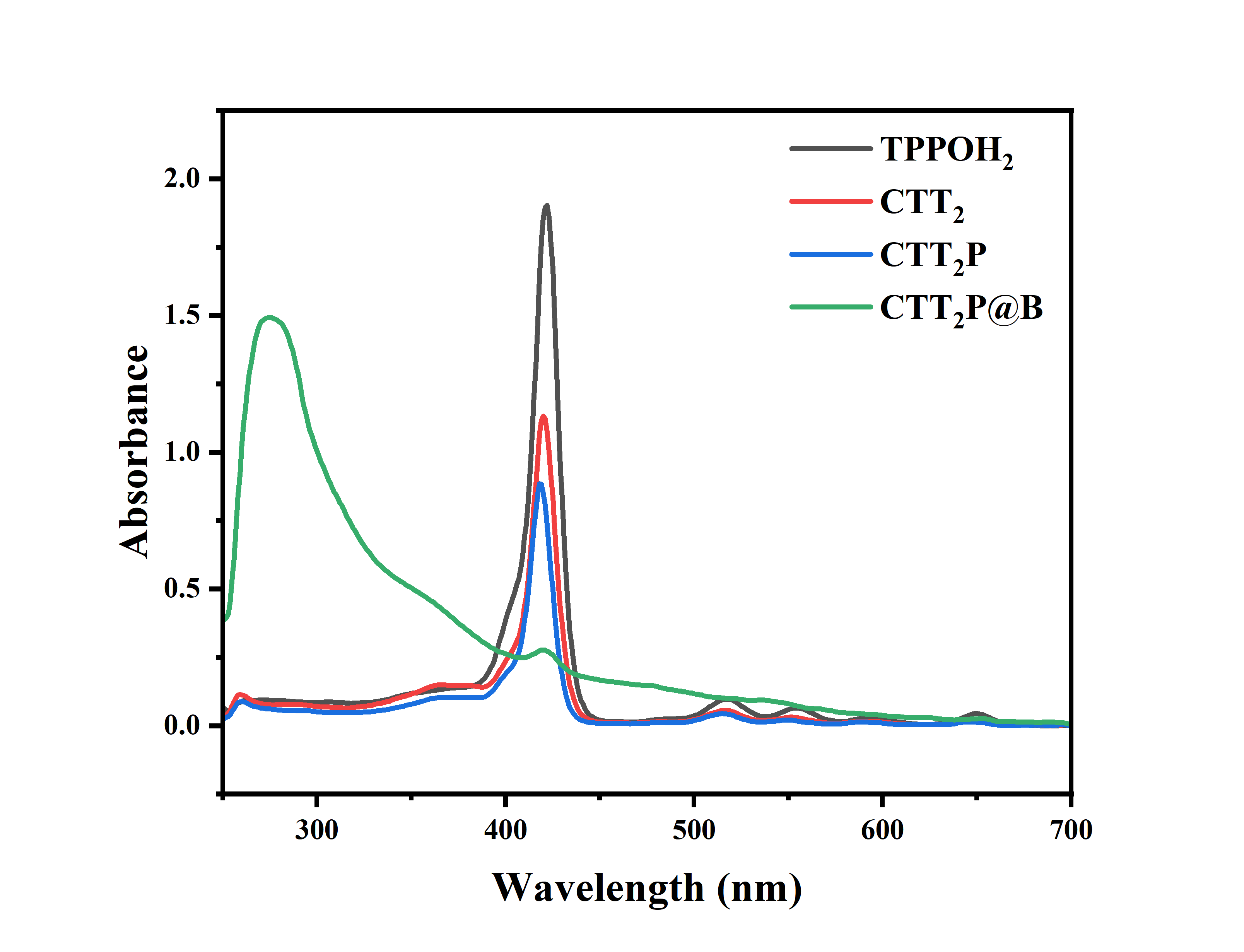


**Supplementary Figure 11.** UV - vis spectra of TPPOH_2_, CTT_2_, CTT_2_P and CTT_2_P@B in DMSO.


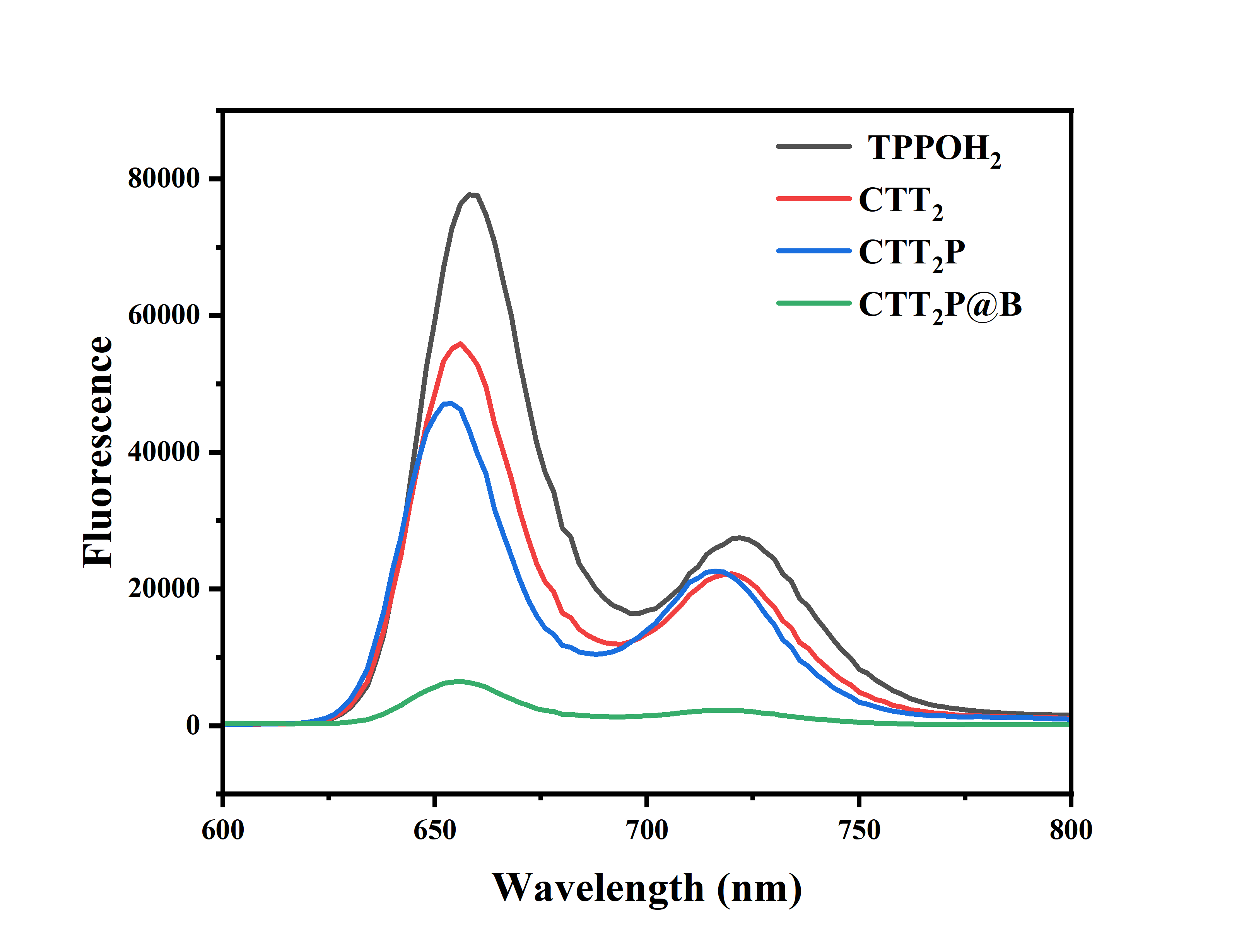


**Supplementary Figure 12.** Fluorescence luminescence spectra of TPPOH_2_, CTT_2_, CTT_2_P and CTT_2_P@B in DMSO; excitation: 420nm.


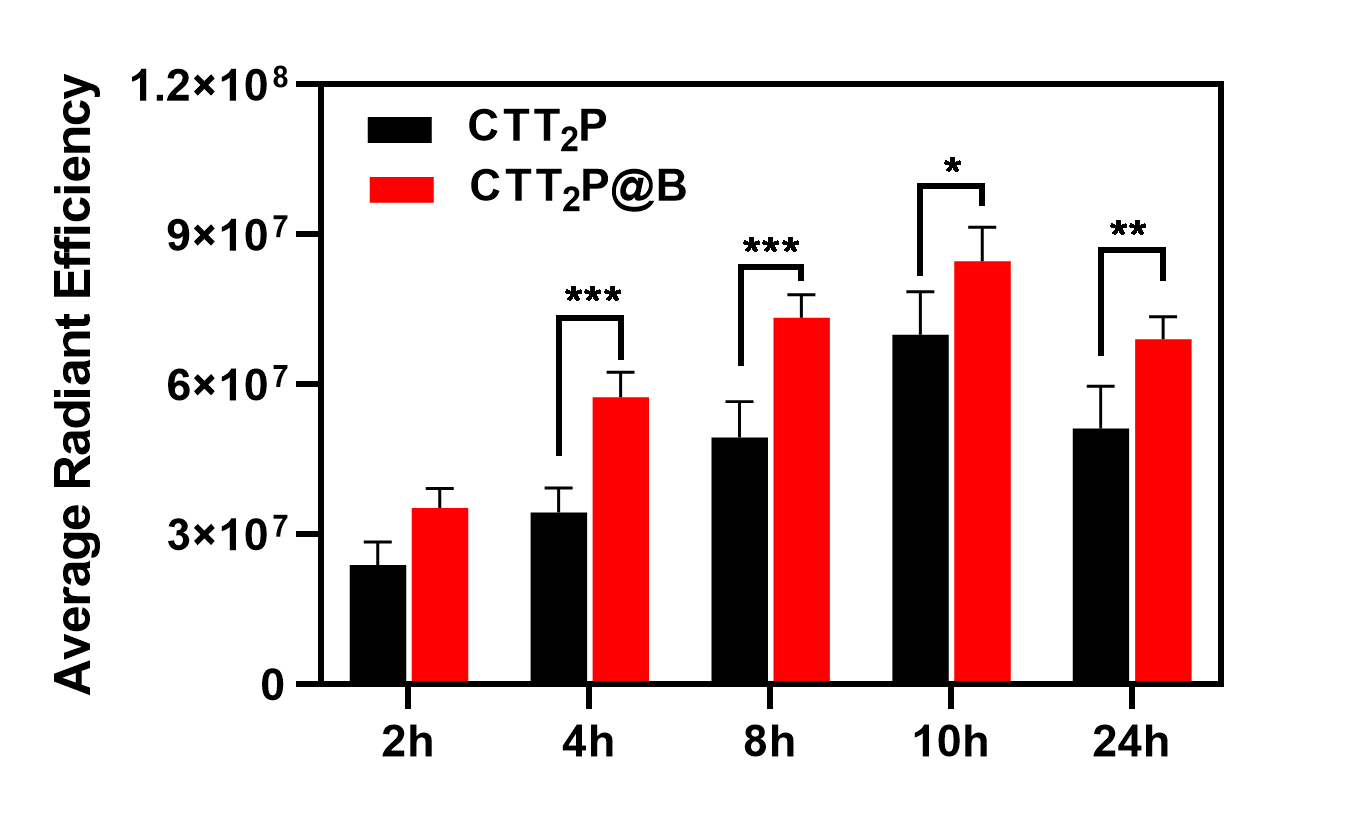


**Supplementary Figure 13.** Time-lapse fluorescence intensity of 4T1 tumor mice after intravenous injection of free CTT_2_P and CTT_2_P@B NPs. Data were presented as the mean ± SD (n = 3). *p <0.05， **p < 0.01, ***p < 0.001.
